# Plasmonic antenna coupling to hyperbolic phonon-polaritons for sensitive and fast mid-infrared photodetection with graphene

**DOI:** 10.1038/s41467-020-18544-z

**Published:** 2020-09-25

**Authors:** Sebastián Castilla, Ioannis Vangelidis, Varun-Varma Pusapati, Jordan Goldstein, Marta Autore, Tetiana Slipchenko, Khannan Rajendran, Seyoon Kim, Kenji Watanabe, Takashi Taniguchi, Luis Martín-Moreno, Dirk Englund, Klaas-Jan Tielrooij, Rainer Hillenbrand, Elefterios Lidorikis, Frank H. L. Koppens

**Affiliations:** 1 ICFO - Institut de Ciències Fotòniques, The Barcelona Institute of Science and Technology, Castelldefels, Barcelona 08860 Spain; 2 Department of Materials Science and Engineering, University of Ioannina, Ioannina, 45110 Greece; 3 Department of Electrical Engineering and Computer Sciences, Massachusetts Institute of Technology, Cambridge, MA 02139 USA; 4 CIC nanoGUNE BRTA, Donostia-San Sebastián, 20018 Spain; 5 Instituto de Ciencia de Materiales de Aragón and Departamento de Física de la Materia Condensada, CSIC-Universidad de Zaragoza, Zaragoza, 50009 Spain; 6 Advanced Materials Laboratory, National Institute for Material Science, Tsukuba, 305-0044 Japan; 7 Catalan Institute of Nanoscience and Nanotechnology (ICN2), Barcelona Institute of Science and Technology, Campus UAB, Bellaterra, Barcelona 08193 Spain; 8 IKERBASQUE, Basque Foundation for Science, Bilbao, 48013 Spain; 9 CIC nanoGUNE BRTA and Department of Electricity and Electronics, UPV/EHU, Donostia-San Sebastián, 20018 Spain; 10 University Research Center of Ioannina (URCI), Institute of Materials Science and Computing, Ioannina, 45110 Greece; 11 ICREA - Institució Catalana de Recerca i Estudis Avançats, Barcelona, 08010 Spain

**Keywords:** Two-dimensional materials, Optical properties and devices, Nanophotonics and plasmonics, Polaritons

## Abstract

Integrating and manipulating the nano-optoelectronic properties of Van der Waals heterostructures can enable unprecedented platforms for photodetection and sensing. The main challenge of infrared photodetectors is to funnel the light into a small nanoscale active area and efficiently convert it into an electrical signal. Here, we overcome all of those challenges in one device, by efficient coupling of a plasmonic antenna to hyperbolic phonon-polaritons in hexagonal-BN to highly concentrate mid-infrared light into a graphene *pn*-junction. We balance the interplay of the absorption, electrical and thermal conductivity of graphene via the device geometry. This approach yields remarkable device performance featuring room temperature high sensitivity (NEP of 82 pW$/\sqrt{{\bf{Hz}}}$) and fast rise time of 17 nanoseconds (setup-limited), among others, hence achieving a combination currently not present in the state-of-the-art graphene and commercial mid-infrared detectors. We also develop a multiphysics model that shows very good quantitative agreement with our experimental results and reveals the different contributions to our photoresponse, thus paving the way for further improvement of these types of photodetectors even beyond mid-infrared range.

## Abstract (web summary)

A significant challenge of infrared (IR) photodetectors is to funnel light into a small nanoscale active area and efficiently convert it into an electrical signal. Here, the authors couple a plasmonic antenna to hyperbolic phonon-polaritons in hexagonal-BN to highly concentrate mid-IR light into a graphene pn-junction.

## Introduction

Hyperbolic phonon-polaritons (HPPs) are hybridized modes of ionic oscillations and light present in polar dielectric materials, such as hexagonal-BN (hBN)^1–7^ that show interesting optical properties such as extreme subwavelength ray-like propagation and sub-diffraction light confinement (~*λ*_0_/100)^1,8–11^, among others. In fact, novel nano-optoelectronic platforms can be attained by merging HPPs functionalities with other 2D materials-based devices, such as graphene photodetectors governed by the photothermoelectric (PTE) effect. This mechanism generates a photoresponse in graphene pn-junctions^12–19^ driven by a temperature gradient and Fermi level asymmetry across the channel. Nevertheless, one of the limitations of these detectors is the low light absorption of graphene, especially for mid-IR frequencies where the photon energy becomes comparable to the typical doping level of graphene reaching the Pauli blocking regime^20,21^. This is further exacerbated by the small photoactive area of graphene *pn*-junctions^16,22^, limited by the cooling length of the hot carriers (0.5–1 *μ*m)^13,14,22,23^. These limitations can be overcome by exciting HPPs and focusing them towards the photoactive area and consequently absorbing them in graphene. However, efficient exploitation of HPPs for mid-IR photodetection still remains unexplored^24,25^. In this work, we embed hBN and graphene within metallic antennas in order to couple their plasmonic interactions with HPPs and achieve highly concentrated mid-IR light on a graphene pn-junction for sensitive and fast mid-IR photodetection.

## Results

### Device operation principle

**Fig. 1:**
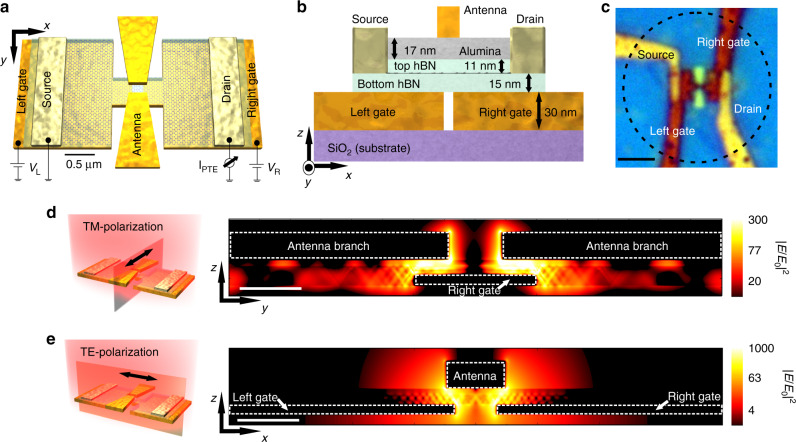
Device schematic and operation principle. **a** Schematic representation of the photodetector consisting of H-shaped resonant gates of 4.2 *μ*m of total length, with a hBN-encapsulated H-shaped graphene channel transferred on top, contacted by source and drain electrodes. A bow-tie antenna of 2.7 *μ*m of total length is placed on top of the 2D stack. The local gates serve to create a *pn*-junction in the central part of the graphene channel (by applying voltages *V*_L_ and *V*_R_), where the antenna gap and gate gap are located. Both narrow gaps are on the order of  ~100 nm. The scale bar corresponds to 0.5 *μ*m. **b** Side view of the device design (not to scale) with indications of the materials' thicknesses. **c** Optical image of the photodetector. The dashed lined circle indicates the typical beam spot size obtained at *λ* =  6.6 *μ*m. The scale bar corresponds to 2.5 *μ*m. **d** Cross section view of the simulated total electric field intensity (∣*E*∣^2^) normalized to the incident one (∣*E*_0_∣^2^) along the antenna main axis when light is polarized parallel to the bow-tie antenna (TM-polarization) axis as indicated in the illustration on the left. The white scale bar corresponds to 250 nm. **e** Same as (**d**) but for light polarization perpendicular to the bow-tie antenna (TE-polarization) and parallel to the local gates as shown in the schematic on the left.

Our design (depicted in Fig. 1a–c) combines several mechanisms to achieve high field concentration for both incident light polarizations. Specifically, when light is polarized parallel to the bow-tie antenna axis (transverse magnetic, TM-polarization, Fig. 1d), it excites its localized surface plasmon resonance (LSPR) spectrally located at *λ* ≈  5–7 *μ*m (Supplementary Figs. 1 and 2a). As a result, the antenna concentrates the incoming mid-IR light into its gap that is situated just above the graphene pn-junction (i.e. the detector photoactive area)^16^. At the same time, the near-fields produced within the antenna hot-spot contain high momenta and thus efficiently launch HPPs ascribed to the spectral overlap of the antenna’s LSPR with the hBN upper reststrahlen band (RB) range (*λ* ≈ 6–7 *μ*m. These HPPs propagate as guided modes and interfere within the graphene pn-junction, producing high absorption across this small localized region. Likewise, when light is polarized perpendicularly to the bow-tie antenna axis (transverse electric, TE-polarization, Fig. 1e), it produces strong light concentration in the gap of the H-shaped antenna, acting as the split-gate, ascribed again to its LSPR spectrally located at *λ* ≈  5.5–7.5 *μ*m (Supplementary Figs. 2b and 3). This phenomenon will also launch hBN HPPs at the gate edges, which will be guided and interfered within the photoactive area.

The absorption process in the graphene is mediated mostly by interband transitions, which mainly occur in the regions within the gap of the gates where the graphene doping is sufficiently small to avoid Pauli blocking (Supplementary Figs. 4 and  5). The excited carriers quickly relax (<100 fs)^23^ into a local hot equilibrium Fermi–Dirac distribution by electron–electron scattering. Subsequent cooling mechanisms include electron–phonon scattering (~1 ps)^13,14,22,23,26^ and heat diffusion away from the junction area. As a result, a symmetric electronic temperature profile *T*_e_(*x*) is produced in the graphene junction^13,14,24^, giving rise to a thermoelectric voltage *V*_PTE_ ∝ *S*(*x*) ∇*T*_e_(*x*), where *x* runs along the graphene channel and *S*(*x*) represents the Seebeck coefficient which is tunable by the gates. Since  ∇*T*_e_(*x*) is antisymmetric, an antisymmetric *S*(*x*) is also needed to maximize the net PTE response, which is achieved by applying opposite voltages to the two gates (Supplementary Figs. 4 and 5). In addition to HPPs promoting absorption in graphene, they also absorb light themselves. However, due to the large (~10^3^) heat capacitance mismatch between graphene electrons and lattice, the HPP absorption does not amount to any meaningful temperature rise and thus does not contribute to the device PTE response.

### Photocurrent characterization and spectral response

**Fig. 2:**
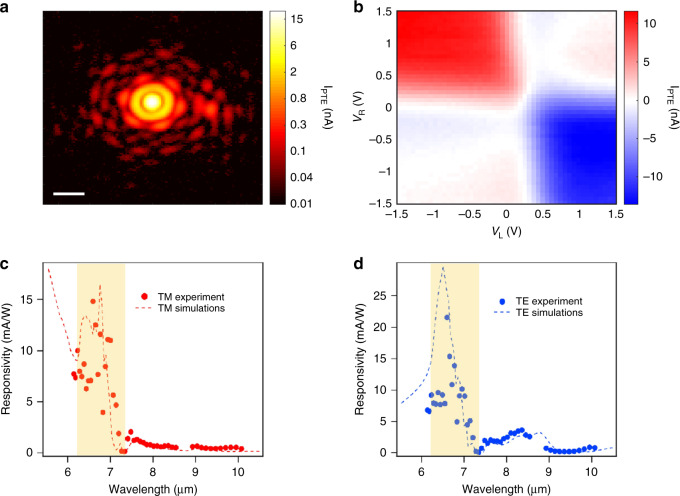
Photocurrent generation and spectral photoresponse. **a** Scanning photocurrent map (log scale) across the mid-IR beam focus at *λ* = 6.6 *μ*m. The white scale bar corresponds to 20 *μ*m. We obtain a FWHM of 6.1 *μ*m. We use a small input power (*P*_in_) of 13.7 *μ*W (irradiance of 0.2 *μ*W/*μ*m^2^). **b** Photocurrent map as a function of the two gate voltages at *λ* = 6.6 *μ*m. **c** Experimental (dots) and theoretical (dashed lines) spectral external responsivity of the device for TM-polarization and (**d**) for TE-polarization. The highlighted region corresponds to the hBN RB (*λ* = 6.2–7.3 *μ*m). For (**c** and **d**), we set the gate voltages to a pn-junction configuration close to the optimal with *V*_L_ = 0.5 V (97 meV) and *V*_R_ = −0.5 V ( −100 meV). We use the same doping level for the theoretical simulations.

To reveal the spatial intensity profile of the beam focus at *λ* = 6.6 *μ*m, we scan the sample with *xyz*-motorized stages and measure the photocurrent (*I*_PTE_) as shown in Fig. 2a. As a result, we observe the Airy pattern of the beam, which implies that we obtain a well-focused beam (see Methods) and high sensitivity at this wavelength considering the small irradiance input of 0.2 *μ*W/*μ*m^2^. Next, we investigate the photoresponse as a function of the two gate voltages (*V*_L_ and *V*_R_), shown in Fig. 2b, which reveals the photocurrent mechanism and optimal doping level. We find that when sweeping the gate voltages independently, the photocurrent follows several sign changes resulting in a 6-fold pattern, which indicates that the photodetection is driven by the PTE effect, as also shown in other studies in the mid-IR range^24,27,28^. The highest values of photocurrent occur at pn or np configuration, specifically at *V*_L_ = 1.6 V (170 meV) and *V*_R_ = −0.82 V (−130 meV), which are relatively low doping levels. We note that when applying a voltage bias in the graphene channel, the photocurrent remains constant while the source-drain current increases linearly with bias (Supplementary Fig. 6). This allows us to discard other mechanisms such as photogating and bolometric effects that would increase significantly with voltage bias.

To determine the photodetector spectral response, we measure the TM-polarization (Fig. 2c) external responsivity (see Methods) as a function of excitation wavelength. We obtain high values up to 15 mA/W within 6–7 *μ*m at the hBN RB. On the other hand, for TE-polarization (Fig. 2d), we observe two responsivity peaks, the first one (up to 22 mA/W) again within the hBN RB (6–7 *μ*m) and a second peak (3.5 mA/W) around 8 *μ*m. We also plot the simulated responsivity that is extracted from the multiphysics simulations, which considers the exact geometry of the photodetector and the whole device photoresponse (optical excitation, carrier distribution and relaxation, heat diffusion and thermoelectric current collection. See further details in Supplementary Notes 1–4). We observe very good qualitative and quantitative agreement between experimental and theoretical responsivity, which we explore in the following by analyzing each component involved in the photoresponse.

### Spectral and spatial analysis of the photoresponse

**Fig. 3:**
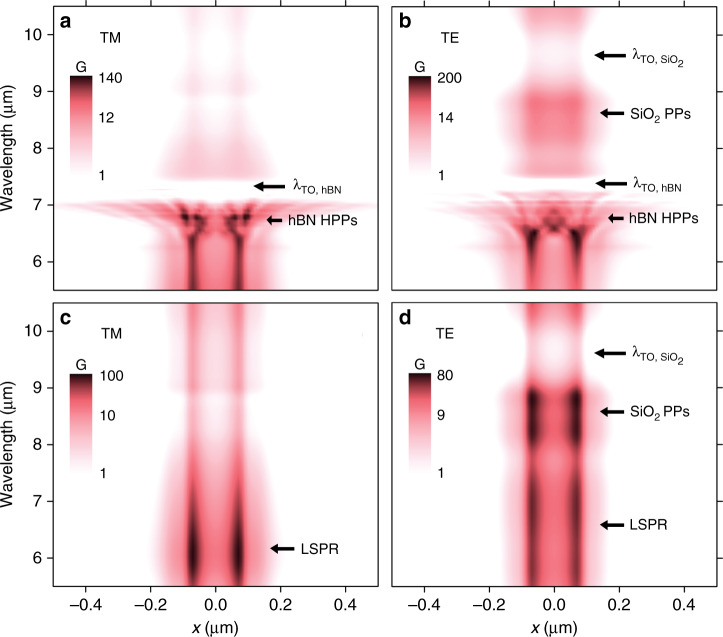
Absorption enhancement spectra. Simulations of the absorption enhancement in graphene (*G*) along the source-drain direction (*x* direction as shown in Fig. 1a, b, where *x* =  0 is located at the center of the gate gap) as a function of the wavelength, for TM (**a**) and TE-polarization (**b**). (**c**) and (**d**) correspond to (**a**) and (**b**), respectively, but with wavelength-independent refractive index for hBN (*n* = 2.4).

We first identify the behavior of the resonant mechanisms, in terms of field intensity enhancement and spatial localization by studying the absorption enhancement in graphene (*G*) across the channel in the *x* direction (averaging over 500 nm in the *y* direction, see Fig. 1a, b for axis definition) and as a function of the wavelength as shown in Fig. 3. We define *G* as follows: *G*(*λ*, **r**) = Abs_device_(*λ*, **r**)/Abs_air_(*λ*, **r**), which is the ratio between the graphene absorption incorporating all the elements of the device (e.g. antenna, contacts, etc.) to that of suspended graphene as a function of *λ* and the position vector **r**. *G* and responsivity are proportionally related via the electronic temperature gradient as shown in Supplementary Fig. 5 and Supplementary Note 2.

In the TM-polarization case shown in Fig. 3a, we observe very high *G* values at the antenna LSPR (*λ* ~ 6 *μ*m). The value of *G* peaks around 6.8 *μ*m due to the hybridization of the hBN HPPs with the antenna LSPR and to the constructive interference of the propagating HPPs occurring at *x* ~ ±100  nm. In fact, the different spatial patterns of *G* arise from the wavelength dependence of the HPP propagation angle in hBN following the equation $\tan \theta (\omega )={\rm{i}}\sqrt{{\varepsilon }_{x,y}(\omega )}/\sqrt{{\varepsilon }_{z}(\omega )}$ (refs. ^1,24,29^). For longer wavelengths, we find a negligible *G* between 7 and 7.3 *μ*m that corresponds to the hBN transverse optical (TO) phonon. We observe that the highest *G* values are only found for the spatially confined region (from *x* ~ −100 to 100 nm) where the antenna and gates overlap, which is designed to coincide with the graphene pn-junction (see Supplementary Figs. 7, 8 and 17–23 regarding the Supplementary Discussion 1). Nevertheless, in the hBN RB we find large *G* values outside this tightly localized region due to HPP propagation.

For TE-polarization (Fig. 3b), we find the maximum values of *G* between 6.2 and 6.6 *μ*m due to the gate LSPR hybridization with HPPs and their strong constructive interference at *x* =  0. For longer wavelengths, we identify a *G* peak centered at 8.5 *μ*m that corresponds to SiO_2_ phonon-polaritons (PPs) hybridization with the gate LSPR as presented in Supplementary Figs. 2 and 9.

To further elucidate the role of the antennas in *G*, we simulate the system without the contribution of the HPPs using wavelength-independent refractive index values for the hBN (Fig. 3c, d). For TM-polarization (Fig. 3c), we observe a peak around 6 *μ*m that corresponds to the antenna LSPR and its resonance tail extending up to 8 *μ*m. For TE-polarization, in contrast, Fig. 3d shows high values of *G* across a broader wavelength range (5.5–7.5 *μ*m) due to the complex shape of the gates and their interactions with the source–drain contacts (Supplementary Fig. 3). Although in Fig. 3d we observe lower *G* values compared to Fig. 3c (Supplementary Fig. 2), we obtain higher values of *G* in TE-polarization when combining the gate LSPR with HPPs (Fig. 3b) ascribed to its higher spectral overlap with the hBN RB and due to the stronger constructive interferences of the HPPs excited by the gates.

**Fig. 4:**
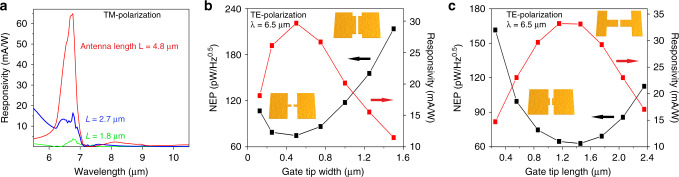
Dependence of the simulated responsivity and NEP on the geometry of the antenna and H-shaped gates. **a** Simulations of responsivity for TM-polarization for different antenna lengths. Different cases are presented: non-resonant antenna within the hBN RB spectral range (antenna total length of *L* = 1.8 *μ*m, shown in green), semi-resonant antenna (*L* = 2.7 *μ*m shown in blue, which corresponds to the experimental antenna) and resonant antenna (*L* = 4.8 *μ*m, shown in red). **b** Simulations of responsivity and NEP as a function of gate tip width and graphene (following the exact shape of the gates) as shown in the schematic for TE-polarization at *λ* = 6.5 *μ*m. The tip length is 855 nm, which includes the gap between the gates of 155 nm. The source-drain distance is 2.6 *μ*m and electrodes width is 2 *μ*m as in the measured device. **c** Same as **b** but as a function of the gate tip length as shown in schematic. The tip width is 500 nm.

To evaluate the coupling between the bow-tie antenna LSPR and the hBN HPPs, we study the responsivity as a function of the antenna length for TM-polarization as shown in Fig. 4a (Supplementary Fig. 10). We observe some hBN HPP excitation when using an antenna non-resonant (green line) within the hBN RB range, in which case we obtain a maximum responsitivity of 4 mA/W. In the case of the semi-resonant antenna (experimental antenna, shown in blue line), whose LSPR partially overlaps with the RB spectral range^25^, the responsivity increases to 17 mA/W, respectively. However, this can be significantly improved if we use a longer antenna (red line) such that its LSPR peak fully overlaps with the hBN HPPs peak, thus obtaining 65 mA/W.

Next, we examine the impact of the H-shaped gates excited at *λ* = 6.5 *μ*m with TE-polarization on the responsivity and NEP (noise-equivalent power, see Methods) by varying the width and length of the gate tip and graphene, while keeping the source-drain distance and width fixed as indicated in Fig. 4b, c. Fig. 4b shows that the responsivity (NEP) increases (decreases) when decreasing the tip width down to an optimal value of 500 nm (same as the experimental value). This is ascribed to the balancing act of absorption, electrical resistance and thermal conductance: larger absorption and lower thermal conductance increase the temperature gradients, but a smaller electrical conductance also reduces the photocurrent and thus the responsivity (Supplementary Fig. 11 and Supplementary Discussion 1). Note that electrical and thermal conductivity are ultimately proportional through the Wiedemann-Franz law. For the case of the gate tip length, however, the optimum is found around 1.45 *μ*m, which is larger than the experimental one (855 nm), pointing to future design and performance improvements (Supplementary Figs. 3 and 11). These results highlight the importance of the gate and graphene channel shapes on PTE performance and the vital role of multiphysics modeling in understanding and optimizing such a complex device.

### Speed, sensitivity, and device benchmark

**Fig. 5:**
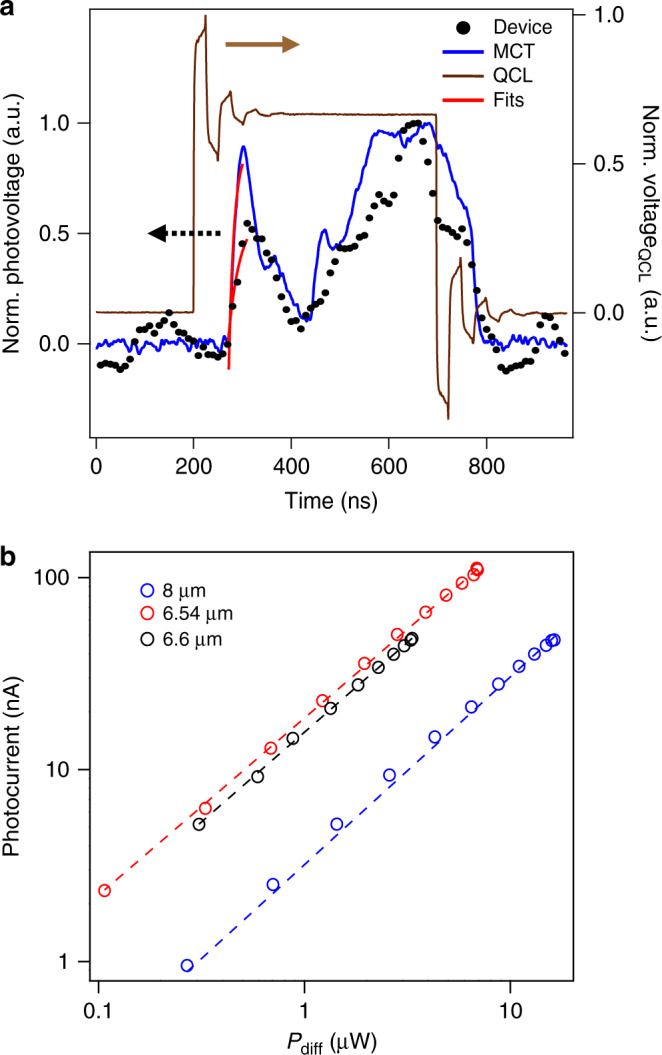
Photodetection speed and power dependence. **a** Time-resolved photodetection traces at *λ* =  6.6 *μ*m, compared with a MCT detector (both plotted in black dots and blue line respectively) and the respective QCL voltage signal (brown line). The QCL pulse width corresponds to 496 ns. The photovoltage fits are shown in red lines. We obtain rise times of 9 ± 3 ns and 17  ±  3 ns for the MCT and our device respectively. **b** Photocurrent as a function of laser power (*P*_diff_ = *P*_in_ × *A*_diff_/*A*_focus_, see Methods) for different wavelengths on a log-log scale. Circles correspond to the data points, while the dashed lines represent the fits according to ${I}_{{\rm{PTE}}}\propto {P}_{{\rm{diff}}}^{\gamma }$. Among all cases, *γ* ranges from 0.92–0.98. We observe linear photoresponse over three orders of magnitude of power (limited by the power meter sensitivity range for *P*_in_ calibration). These results suggest that we are operating in the weak heating regime (*T*_e_ − *T*_l_ << *T*_l_)^16,23^, as in the strong heating regime (*T*_e_ − *T*_l_ >> *T*_l_), a sublinear behavior is expected (*γ* =  0.5)^16,23^. Here *T*_e_ is the electronic temperature and *T*_l_ is the graphene lattice temperature, which the latter is in thermal equilibrium with the environment.

Now we discuss the technological relevance of our photodetector. First, we measure the photodetection speed by using as reference a commercial fast mercury–cadmium–telluride (MCT) detector. We plot in Fig. 5a the quantum cascade laser (QCL) voltage (brown line) together with the photoresponses of the MCT (blue line) and our device (black circles). The signal of the MCT detector reveals the pulse shape of the laser. We fit an exponential function to the initial peak to determine the rise time (shown in red lines), obtaining a value of 9.5 ns, which is close to its datasheet value of 4.4 ns. In the case of our photodetector, we find a rise time of 17 ns (22 MHz) when using a current amplifier with 14 MHz bandwidth. This suggests that our time-resolved measurements are limited by the current amplifier bandwidth (Supplementary Fig. 12), meaning that the actual rise time may be shorter. In fact, our theoretical calculations predict a speed of 53 ps (Supplementary Note 5).

The sensitivity of the detector is best expressed in terms of external responsivity, which the maximum measured value is 27 mA/W (92 V/W, Supplementary Fig. 13), yielding a noise-equivalent-power of 82 pW/$\sqrt{{\rm{Hz}}}$ (refs. ^24,30–33^), assuming the graphene thermal noise as the dominating noise source^16,34,35^. We emphasize that the zero-bias operation leads to low noise levels and a very low power consumption, which is given by the voltage applied to the gates. Furthermore, our design allows sensitive detection in different polarizations (Supplementary Fig. 14), which is a limitation for the mentioned graphene detectors^24,30–32^. Additionally, our device exhibits a wide dynamic range by showing linear photoresponse over three orders of magnitude as shown in Fig. 5b, which is an issue for other types of graphene detectors^31^ and commercial detectors such as MCT^36^. It also has a very small active area given by the antennas’ cross-sections, which implies high spatial resolution and opens the possibility of arranging it into high density photodetector pixels^30,37^ that are CMOS compatible^38^. All of these performance parameters combined make our device an interesting platform that fulfills the ongoing trend of decreasing the size, weight and power consumption (SWaP) of infrared imaging systems^36^.

## Discussion

The device concept introduced in this work can be extended to detectors for other wavelengths or more specific functionalities such as hyperspectral imaging and spectroscopy. Our approach can also be combined with HPPs in other regions of the mid-IR and long-wave infrared range such as MoO_3_ (refs. ^39–41^). Additional tuning and wavelength sensitivity can be realized by controlling the hyperbolic material’s thickness^24,42^ or shape^1,43–45^.

## Methods

### Device fabrication

First, we fabricate the H-shaped local gates structure with a total length of 4.2 *μ*m, a total width of 2 *μ*m and a narrow width region (tip) of 500 nm on a Si/SiO_2_ substrate using electron beam lithography (EBL) followed by evaporation of titanium (2 nm)/gold (30 nm). The gap between the gates is 155 nm. Afterwards, we transfer a hBN/graphene/hBN stack onto the metallic gates. We cleave and exfoliate the top and bottom hBN and the graphene onto freshly cleaned Si/SiO_2_ substrates, stack them following the Van der Waals assembly technique^46,47^ and release onto the gates. We then use EBL with a PMMA 950 K resist film to pattern source and drain electrodes and expose the device to a plasma of CHF_3_/O_2_ gases to partially etch the Van der Waals stack. Subsequently, we deposit side contacts of chromium (5 nm)/gold (80 nm) and lift off in acetone as described in ref. ^46^. We then etch the hBN-encapsulated graphene into an H-shape using a CHF_3_/O_2_ plasma and deposit 17 nm of Al_2_O_3_ using atomic layer deposition (ALD). Finally we pattern the bow-tie antenna of 2.7 *μ*m total length (*L*) and with a small gap of 200 nm between its branches with EBL and deposit titanium (2 nm)/gold (80 nm). We point out that the bow-tie antenna and gate dimensions were selected based on preliminary optical simulations based on a simplified device, ignoring bow-tie antenna interactions with gates and metal electrodes, resulting in the non-optimal performance as evident from Fig. 4. By performing 2-terminal configuration electrical measurements as a function of the gate voltages (varying *V*_L_ and *V*_R_ both at the same potential), we attain 12,000 cm^2^ V^−1^ s^−1^ as a lower bound of the estimated mobility (Supplementary Figs. 15, 16 and Supplementary Note 4).

### Measurements

We use a pulsed QCL mid-IR laser (LaserScope from Block Engineering) that is linearly polarized and has a wavelength tuning range from *λ* = 6.1 to 10 *μ*m. We scan the device position with motorized *xyz*-stage. We modulate the mid-IR laser employing an optical chopper at 422 Hz and we measure the photocurrent using a lock-in amplifier (Stanford Research). We focus the mid-IR light with a reflective objective with a numerical aperture (NA) of 0.5. We measure the mid-IR power using a thermopile detector from Thorlabs placed at the sample position.

For the time-resolved measurements, we set the QCL wavelength to *λ* = 6.6 *μ*m with a pulse width of 496 ns. We use a MCT as a reference detector from VIGO System model PCI-2TE-13. We measure the photoresponse using a current amplifier from FEMTO model DHPCA-100 with switchable gain and acquire the signal with an oscilloscope from Teledyne Lecroy model HDO6000.

### Responsivity and NEP calculation

The external responsivity is given by: Responsivity = (*I*_PTE_/*P*_in_) × (*A*_focus_/*A*_diff_)^16,34,48,49^, where *P*_in_ is the power measured by the commercial power meter, *A*_focus_ is the experimental beam area at the measured wavelength and *A*_diff_ is the diffraction-limited spot size. We measure the photocurrent *I*_PTE_ from the output signal of the lock-in amplifier *V*_LIA_ considering ${I}_{{\rm{PTE}}}=\frac{2\pi \sqrt{2}}{4\xi }{V}_{{\rm{LIA}}}$ (refs. ^34,48,49^), where *ξ* is the gain factor in V/A (given by the lock-in amplifier). We use the ratio *A*_diff_/*A*_focus_ for estimating the power reaching our photodetector since *A*_diff_ is the most reasonable value one can attain when considering the detector together with an optimized focusing system (e.g. using hemispherical lens) and it is widely used in the literature for comparing the performances among photodetectors^16,34,48,49^. Note that this simple geometrical scaling is not used here to predict the exact performance at a hypothetical diffraction-limited spot but rather as clear performance benchmark so to consistently compare different device architectures measured at differed focusing conditions. We usually have a ratio of *A*_focus_/*A*_diff_ ≈ 7. This ratio is given by ${A}_{{\rm{diff}}}/{A}_{{\rm{focus}}}=\frac{{w}_{0,{\rm{diff}}}^{2}}{{w}_{0,{\rm{x}}}{w}_{0,{\rm{y}}}}$. In order to obtain *w*_0,x_ and *w*_0,y_ we use our experimental observation that the photocurrent is linear in laser power and measure the photocurrent while scanning the device in the *x-*  and *y-*direction. Consequently, the photocurrent is described by Gaussian distributions $\propto {{\rm{e}}}^{-2{x}^{2}/{w}_{0,{\rm{x}}}^{2}}$ and $\propto {{\rm{e}}}^{-2{y}^{2}/{w}_{0,{\rm{y}}}^{2}}$, where *w*_0,x_ and *w*_0,y_ are the respectively obtained spot sizes (related to the standard deviation via *σ* = *w*_0_/2 and to the FWHM = $\sqrt{2\mathrm{ln}\,(2)}{w}_{0}$). We usually achieve *w*_0,x_ = 5.05 *μ*m and *w*_0,y_ = 5.40 *μ*m at *λ* = 6.6 *μ*m (Supplementary Fig. 13b). For the diffraction-limited spot, we consider ${w}_{0,{\rm{diff}}}=\frac{\lambda }{\pi }$, with *λ* the mid-IR laser wavelength. The diffraction-limited area is hence taken as ${A}_{{\rm{diff}}}=\pi {w}_{0,{\rm{diff}}}^{2}={\lambda }^{2}/\pi$. Additionally, the noise-equivalent power (NEP) that characterizes the sensitivity of the photodetector is defined as NEP  = *I*_noise_/Responsivity and considering that our unbiased photodetector has a very low noise current that is limited by Johnson noise, we use a noise spectral density ${I}_{{\rm{noise}}}=\sqrt{\frac{4{k}_{{\rm{B}}}T}{{R}_{{\rm{D}}}}}$, where *k*_B_ corresponds to the Boltzmann constant, *T* is the operation temperature (300 K) and *R*_D_ the device resistance.

## Supplementary information

Supplementary Information

## Data Availability

The data that support the plots within this paper and other findings of this study are available from the corresponding author upon reasonable request.
