# Supplementary material for: Plasmonic antenna coupling to hyperbolic phonon-polaritons for sensitive and fast mid-infrared photodetection with graphene
Source: Nat Commun. 2020 Sep 25;11:4872. doi: 10.1038/s41467-020-18544-z (PMC7519130; doi:10.1038/s41467-020-18544-z)
Supplement: Supplementary file 1 — Supplementary Information [file 41467_2020_18544_MOESM1_ESM.pdf]

Supplementary Information:
Plasmonic antenna coupling to hyperbolic phonon-polaritons for sensitive and fast mid-infrared photodetection with graphene

S. Castilla et al.

SUPPLEMENTARY NOTE 1: OPTICAL MODELING

We perform optical calculations with the full vector 3D finite-difference time domain (FDTD) method using Lumerical software. The computational cell dimensions are 6×6×6 µm^2 with perfectly matched layer (PML) conditions employed on all boundaries. We use a grid of 5 nm in lateral (x,y) and 2 nm in vertical (z) directions. The light source is modeled as an incident plane wave reaching the device through an open aperture of 5.9×5.9 µm^2 with spectral range 5.5-10.5 µm. The dimensions of the device layers are described in the *Fabrication* section in Methods.

We fit the Au dielectric function using the Drude model $\varepsilon(\omega) = \varepsilon_\infty - \omega_P^2/(\omega^2 + i\Gamma\omega)$ with ω_P the plasma frequency and Γ the plasma collision rate. hBN is optically anisotropic [1], with different permittivities along the in-plane ($\perp c$) and out-of-plane ($\| c$) directions. We fit both using the Lorentz model $\varepsilon(\omega) = \varepsilon_\infty + s\omega_0^2/(\omega_0^2 - \omega^2 - i\gamma\omega)$, where s is a dimensionless coupling factor, ω_0 the normal frequency of vibration and γ the decay rate amplitude. Supplementary Table 1 shows the parameters for Au and hBN. The refractive indices used for SiO_2 and Al_2O_3 are taken from literature[2], while for Si we use $n_{Si} = 3.42$. Graphene is implemented as a 2D surface with optical response modeled by the Kubo conductance[3] $\sigma = \sigma_{intra} + \sigma_{inter}$, where:

$$\sigma_{intra} = \frac{ie^2}{\pi\hbar^2\Omega}\int_0^\infty \epsilon(\partial_\epsilon f(-\epsilon;\mu,T_e) - \partial_\epsilon f(\epsilon;\mu,T_e))d\epsilon \tag{1}$$

$$\sigma_{inter} = \frac{ie^2\Omega}{\pi\hbar^2}\int_0^\infty \left(\frac{f(-\epsilon;\mu,T_e) - f(\epsilon;\mu,T_e)}{\Omega^2 - 4(\epsilon/\hbar)^2}\right)d\epsilon \tag{2}$$

Here Ω is defined as $\Omega = \omega + i\tau_{opt}^{-1}$, $\tau_{opt} = 200$ fs is the assumed electron relaxation time, $f(\epsilon;\mu,T_e) = [e^{(\epsilon-\mu)/k_B T_e} + 1]^{-1}$ is the Fermi-Dirac distribution and $\partial_\epsilon = \partial/\partial\epsilon$.

**Material**	ε_∞	**s**	ω_0, ω_P **[eV]**	γ_0, Γ **[eV]**
hBN[1] $\perp c$	4.87	1.83	0.17	0.87
hBN[1] $\| c$	2.95	0.61	0.0925	0.25
Au[4]	10.78	-	9.13	0.07

Supplementary Table I: Dielectric permittivity parameters of Au and hBN.

SUPPLEMENTARY NOTE 2: THERMOELECTRIC MODELING

We assume the quasi-continuous-wave case and solve the heat dissipation equation[5]:

$$-\nabla\cdot(\kappa\nabla T_e) = \nabla\Pi\cdot\mathbf{j}_q - \tau_{e-ph}^{-1}c_e\Delta T + \alpha P_{dens} \tag{3}$$

where κ is the electronic thermal conductivity, T_e the electronic temperature, $\Pi = ST_e$ the Peltier coefficient, S the Seebeck coefficient, $\mathbf{j}_q = -\sigma S\nabla T_e$ the local thermoelectric current, σ the electrical conductivity, τ_{e-ph} the average cooling time (3 ps), c_e the electronic heat capacity, $\Delta T = T_e - T_l$, T_l is the lattice temperature, α the absorption fraction and the incident power density $P_{dens} = P_{in}/A_{diff}$, where P_{in} is the source power and $A_{diff} = \lambda^2/\pi$ is the diffraction-limited area. The term c_e/τ_{e-ph} in Supplementary Equation 3 is equivalent to Γ_{cool}, where Γ_{cool} ($\sim$ 4-5×10^4 W/Km^2) is the interfacial heat conductivity[6,7]. Due to the large lattice heat capacity (compared to the electronic one) we assume constant $T_l = 300$ K. The graphene parameters σ, S, c_e, κ, Γ_{cool} and α are functions of position, depending on both the local Fermi level E_F and the local temperature T_e, making this a highly non-linear problem. The self-consistent solution of Supplementary Equation 3 provides the T_e distribution from which the thermoelectric voltage is obtained:

$$V_{PTE} = W^{-1}\int_0^W\int_0^L S\nabla T_e dxdy \tag{4}$$

where the length L is the distance between the contacts (assumed in the x direction, as shown in Fig. 1a-b in the main text) and W the width of the graphene channel (y direction, as shown in Fig. 1a-b). The photocurrent

is then $I_{\rm PTE} = V_{\rm PTE}/R_{\rm D}$, where $R_{\rm D} = R_{\rm g} + 2R_{\rm c}$ with $R_{\rm g}$ is the resistance of graphene channel $R_{\rm g} = \int_0^L \sigma^{-1}(x)dx$, $\sigma(x) = \int_0^W \sigma(x,y)dy$ and $R_{\rm c}$ the contact resistance of the device. The responsivity and NEP are calculated following the description in *Responsivity and NEP calculation* section in Methods.

Most parameters in the above equations depend on the local Fermi level and electronic temperature. The former is obtained from the graphene charge density $E_{\rm F} = \hbar v_{\rm F}\sqrt{\pi n^{(0)}}$, where $n^{(0)}$ is calculated by electrostatic simulations (see Supplementary Note 3 and Supplementary Figure 4) using the ratio $\epsilon_{\rm hBN}/d_{\rm hBN}$ as determined by fitting the measured device resistance (see Supplementary Note 4). At finite temperature we obtain the chemical potential from the solution of $\int_0^\infty v(\epsilon)f(\epsilon;\mu,T_{\rm e}) - f(\epsilon;-\mu,T_{\rm e})d\epsilon = E_{\rm F}^2/\pi\hbar^2 v_{\rm F}^2$, where $v(\epsilon) = \frac{2|\epsilon|}{\pi\hbar^2 v_{\rm F}^2}$ is the graphene density of states at energy ϵ and $v_{\rm F} = 1\times10^6$ m/s the graphene Fermi velocity. The rest of the graphene electrical and thermal parameters are calculated as follows:

<u>Electrical conductivity:</u>
$\sigma(\mu,T_{\rm e}) = \int_{-\infty}^{\infty}\sigma(\epsilon)\partial_\epsilon f(\epsilon;\mu,T_{\rm e})d\epsilon$, where $\sigma(\epsilon) = q[\mu_{\rm q} n(\epsilon) + \bar{\mu} n^*(\epsilon)]$, with charge carrier mobility $\mu_{\rm q} = \mu_{\rm e}(\mu_{\rm h})$ for $\epsilon > 0(\epsilon < 0)$ and $\bar{\mu} = (\mu_{\rm e} + \mu_{\rm h})/2$. The effective residual local charge fluctuation at energy ϵ is assumed to be $n^*(\epsilon) = \sqrt{n(\epsilon)^2 + n_0^{*2}} - n(\epsilon)$, where $n(\epsilon) = \frac{\epsilon^2}{\pi\hbar^2 v_{\rm F}^2}$ is the graphene charge density at energy ϵ and n_0^* is the residual local charge fluctuations in the charge neutrality point[8,9].
<u>Seebeck coefficient</u> is given by the general Mott formula[10]:
$S(\mu,T_{\rm e}) = -(|e|T_{\rm e}\sigma)^{-1}\int_{-\infty}^{\infty}(\epsilon-\mu)\sigma(\epsilon)\partial_\epsilon f(\epsilon;\mu,T_{\rm e})d\epsilon$
<u>Thermal capacity[11]:</u>
$c_{\rm e}(\mu,T_{\rm e}) = \partial_{T_{\rm e}}\int_0^\infty(v(\epsilon)\epsilon[f(\epsilon;\mu,T_{\rm e}) + f(\epsilon;-\mu,T_{\rm e})]d\epsilon$
<u>Thermal conductivity:</u> is given by the Wiedemann-Franz law $\kappa(\mu,T_{\rm e}) = L_0\sigma(\mu,T_{\rm e})T_{\rm e}$, where $L_0 = 2.44\times10^{-8} W\Omega K^{-2}$ is the Lorenz number.

SUPPLEMENTARY NOTE 3: ELECTROSTATIC MODELING

The surface charge density of the graphene sheet is calculated by solving the Poisson equation after applying the appropriate voltages $V_{\rm L}$ and $V_{\rm R}$ at the two branches of the split gate. The graphene channel is introduced as a grounded surface above the hBN dielectric spacer layer. The nearby metal contacts are also set to ground. The dielectric constants of Si, SiO_2 and hBN are set to 11.7, 3.9 and 3.5[12–14] respectively. The surface charge density is calculated for both symmetric and anti-symmetric gating cases (see Supplementary Figure 4a).

SUPPLEMENTARY NOTE 4: DEVICE RESISTANCE MODELING

The measured device resistance $R_{\rm D}$ as a function of symmetric gate voltage ($V_{\rm L}$=$V_{\rm R}$) is fitted considering $R_{\rm D} = R_{\rm g} + 2R_{\rm c}$ and with fitting parameters the electron-hole mobilities, the residual local charge fluctuations n_0^*, the value of $\varepsilon_{\rm hBN}/d_{\rm hBN}$, the Dirac voltage $V_{\rm D}$ (for charge neutrality) and the contact resistance $R_{\rm c}$. The charge density distribution is obtained by the electrostatic calculations. We obtain $\mu_{\rm e}(\mu_{\rm h})$ =10,200 (11,900) cm^{2}V^{-1}s^{-1}, $n_0^* = 1.72\times10^{11}$ cm^{-2}, $V_{\rm D} = 0.108$ V and $\varepsilon_{\rm hBN}/d_{\rm hBN} = 0.222$ nm^{-1}. From the latter we extract hBN thickness $d_{\rm hBN} = 15.7$ nm, in excellent agreement with the 15 nm used in this work, validating our approach. The contact resistance is described with a Gaussian distribution around the charge neutrality point[15], with $R_{\rm c} = 0.2$ (0.9) kΩ at high (low) doping (see Supplementary Figure 16).

SUPPLEMENTARY NOTE 5: SPEED CALCULATIONS

The operation frequency of the photodetector is intrinsically related to the RC-time constant $\tau = R_{\rm D}C_{\rm D}$, with $R_{\rm D}$ as the total device resistance (see Supplementary Note 2 and 4) and $C_{\rm D}$[16] as the total device capacitance given by $C_{\rm D}^{-1} = C_{\rm q}^{-1} + C_{\rm G}^{-1}$, where $C_{\rm q}$ is the quantum graphene capacitance and $C_{\rm G}$ is the capacitance of the system as given by the normal formula for parallel-plate capacitors (for the case where $V_{\rm L} = V_{\rm R} = 0.5$ V and $\varepsilon_{\rm hBN}/d_{\rm hBN} = 0.222$ nm^{-1} as described in Supplementary Note 3 and 4). The operating speed is then described by the rate $f = (2\pi\tau)^{-1}$ and the rise time $\tau_{\rm rise}$, which is the time required for the photodetector to increase its output signal from 10% to 90% of the final steady-state output level. The rise time is calculated as $\tau_{\rm rise} = \tau\times\ln(9) = (2\pi f)^{-1}\cdot\ln(9) = 0.35/f$.

SUPPLEMENTARY FIGURES

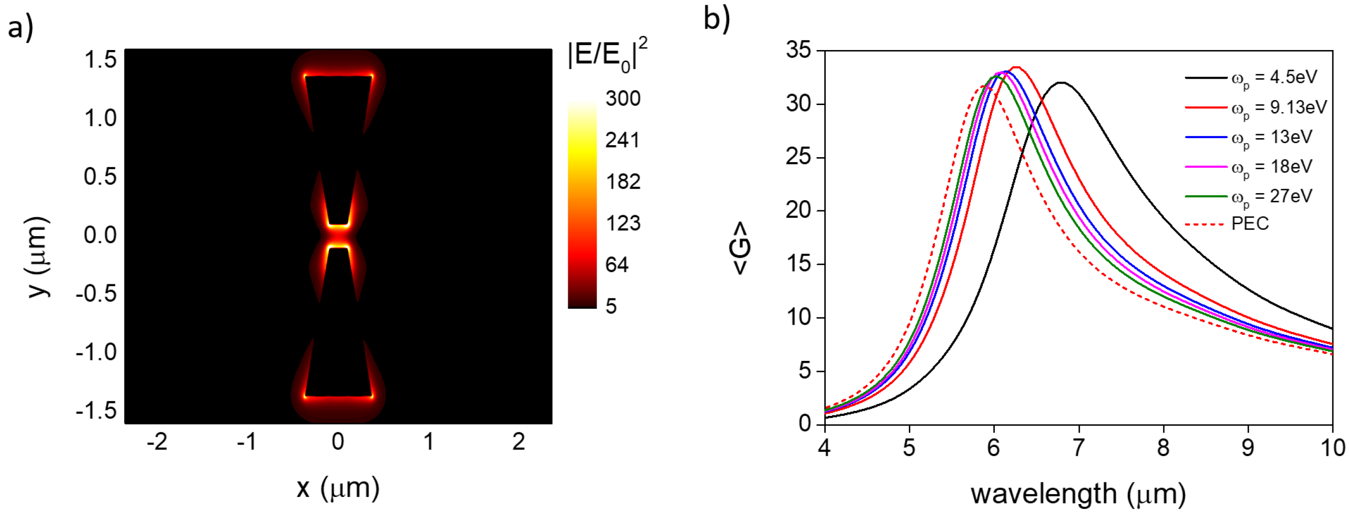

Supplementary Figure 1: a) Simulated electric field intensity enhancement for light polarized parallel to the bow-tie antenna main axis (TM-polarization) at $\lambda\ = 5.5$ μm for the full experimental structure described in the main text. The antenna exhibits a dipole field distribution with high field confinement in the antenna gap and small field penetration inside the metal. **b)** Average absorption enhancement (G, as defined in the main text) within a 0.2×0.2 μm^2 area at the device center ($x = y = 0$, see also Fig. 1a-b in the main text for axis definition). For simplicity, we use wavelength-independent refractive indices for the other materials $n_{hBN} = 2.4$, $n_{SiO_2} = 1.5$ and $n_{Al_2O_3} = 1.6$. Different antenna metals are considered in the Drude model by assuming different plasma frequencies (9.13 eV for Au). We observe that the response changes in both amplitude and spectral position for different plasma frequencies and is different from the PEC case (to which they eventually converge for increasing plasma frequency). We conclude that while the Au plasmons do not directly influence the device physics, they do modulate the antenna response and are thus needed for an accurate quantitative description of the metal antenna response.

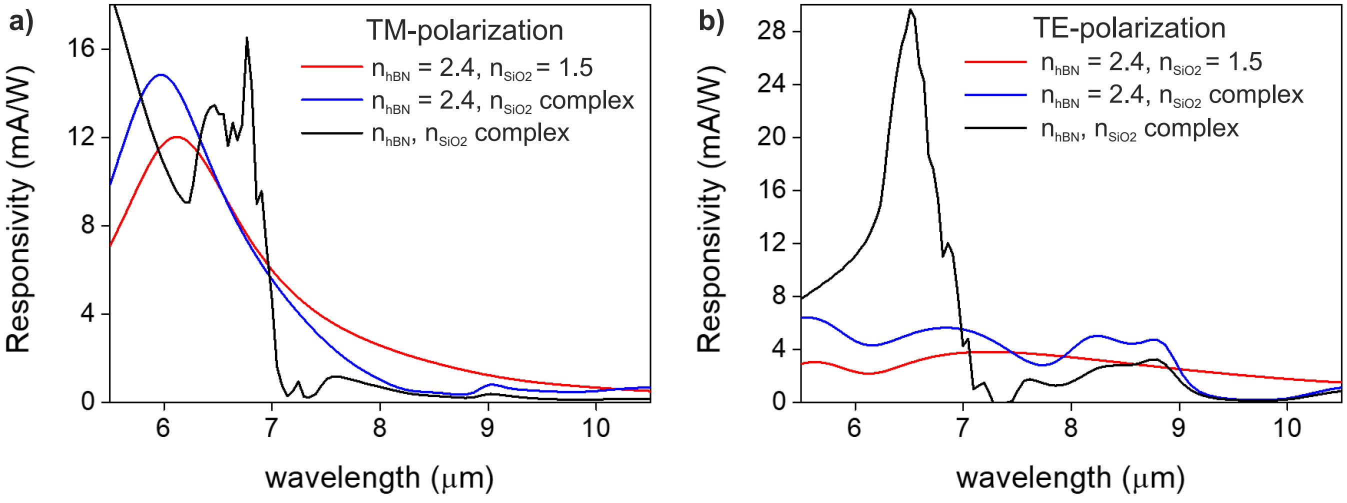

Supplementary Figure 2: Simulated spectral responsivities of **a)** TM and **b)** TE-polarization using different refractive indices configurations. Red solid line represents the case of wavelength-independent refractive indices for hBN ($n = 2.4$), SiO_2 ($n = 1.5$) and alumina ($n = 1.6$). For blue solid line we use the full dispersive optical model for the SiO_2 but not for the hBN, for which we use a wavelength independent refractive index of $n = 2.4$. Finally, for the black solid line we use full dispersive optical model for both SiO_2 and hBN.

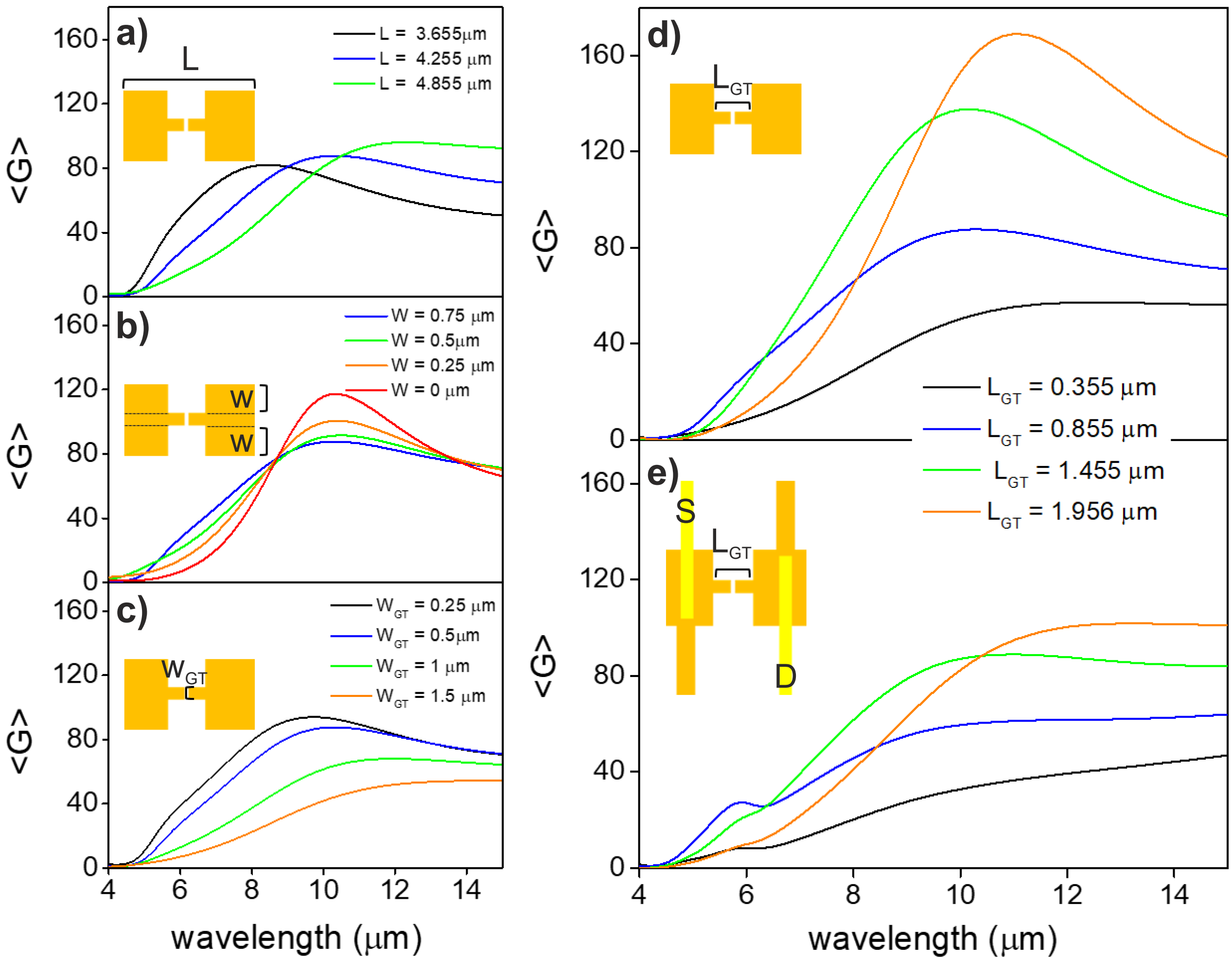

Supplementary Figure 3: Simulated $<G>$, which is G averaged over the 0.2×0.2 μm^2 rectangular space around the center of the device ($x = 0$, $y = 0$, see Supplementary Figure 1a and Fig. 1a-b in the main text for axis definition), for TE-polarization as a function of the geometrical parameters of the H-shaped gates and additional metal components for the spectral range from 4 to 15 μm. In all cases, we use wavelength-independent refractive indices for hBN ($n = 2.4$), SiO_2 ($n = 1.5$) and alumina ($n = 1.6$), hence we can clearly observe the plasmonic response of the local gates without additional resonances coming from the phonon-polaritons of hBN or SiO_2. In all cases the blue line corresponds to the response of the configuration where the varied parameter has the same value as the one it has in the experimental device. **a)** $<G>$ values for different total lengths (L) of the local gates. We observe resonant peaks for all cases which shift to longer wavelengths as we increase L, a typical trend of metallic plasmonic resonators. In **b)** the width (W) of both extended parts of the H-shaped local gates is reduced starting from 0.75 μm (blue line) down to 0 μm (red line) while keeping L fixed at 4.255 μm. We clearly observe that the resonance peak is not shifted spectrally and also as W is reduced the spectral response converges to that of a dipole antenna (red line), thus proving the plasmonic behavior of the local gate geometry. In **c)**, we vary the gate tip width (W_{GT}) and notice that when increasing it, its plasmonic resonance is redshifted, its $<G>$ amplitude drops down and the resonance becomes broader. In **d)** we vary the length of the gate tip (L_{GT}) while keeping L and W fixed at 4.255 μm and 0.75 μm respectively. We observe that the plasmonic response can be strongly tuned both spectrally and in amplitude when changing L_{GT}. Finally, in **e)** we add the extended electrodes to the local gates and also the source-drain contacts. We observe that these additional metal components further alter the response which is now lowered and broadened while we also notice an extra resonance peak at 5.8 μm. Note also that in the wavelength range of the hBN upper RB (6-7 μm) the optimum L_{GT} should be around 1.4 μm as shown in **d** and **e**. Due to the complexity of the H-shape configuration there are a large number of parameters that can strongly tune the plasmonic resonance of the local gates, meaning that we can further improve the device performance.

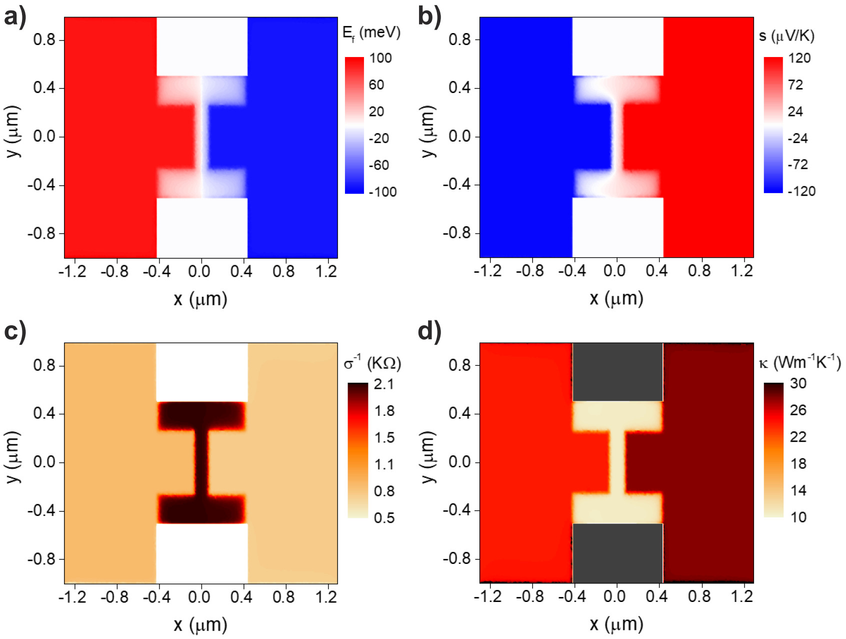

Supplementary Figure 4: Electrical and thermal properties of the graphene channel when applying 0.5 V (-0.5 V) to the left (right) gate. **a)** Calculated Fermi energy for the *n*-doped region (above left gate region) and *p*-doped region (above right gate region). In the gate gap, which is 155 nm, the Fermi energy drops to zero as we move from the *n*-doped to the *p*-doped gated region. Low values of the Fermi energy are also present in the graphene patches that extend 250 nm above and below the gate tips. **b)** Calculated Seebeck coefficient of the different graphene regions. As with the Fermi energy, the Seebeck coefficient drops to zero in the gate gap. Due to the difference in electron and hole mobilities, an imbalance is present in the weakly doped regions. Calculated **c)** resistivity and **d)** thermal conductivity. These two parameters are inversely proportional via the Wiedemann-Franz law.

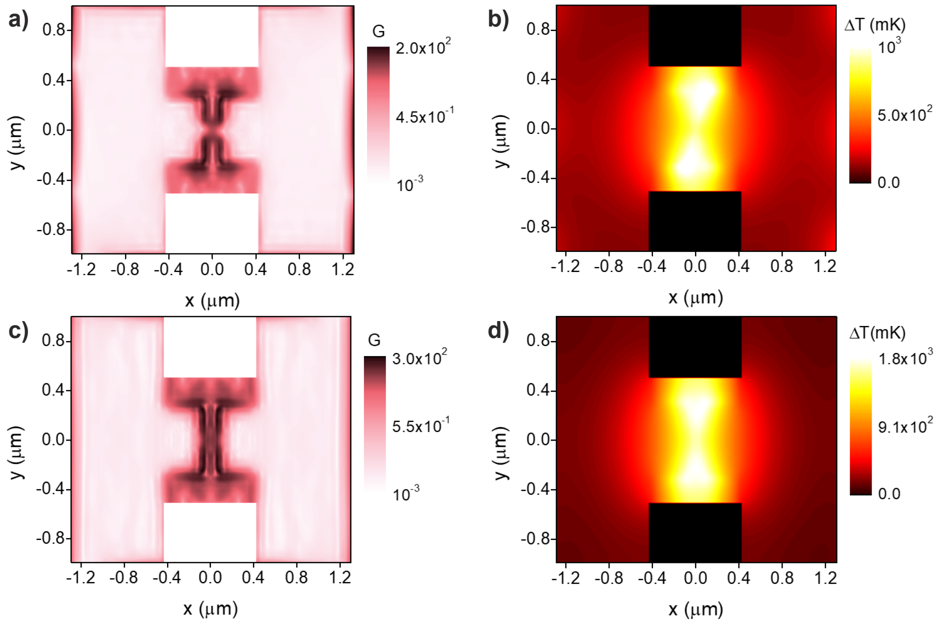

Supplementary Figure 5: **a)** G values in the graphene channel and **b)** temperature distribution for TM-polarization and **c)** and **d)** for TE-polarization respectively. For both cases the incident wavelength is 6.5 µm. In both polarizations we observe the maximum values of G in an area of $0.4{\times}0.5$ µm^2 around the center of the *pn*-junction (x=0, y=0). The fact that in the rest of the graphene channel G values are 3-4 orders of magnitude lower compared to the ones above indicates efficient light focusing in the *pn*-junction. This enhanced absorption and low thermal conductivity (shown in Supplementary Figure 4d) across the *pn*-junction results in high temperature concentration, as is evident in **b** and **d**. Note that the difference in G values for the two cases corresponds to a difference in peak temperatures and finally in difference a responsivity as shown in Fig. 2 in the main text.

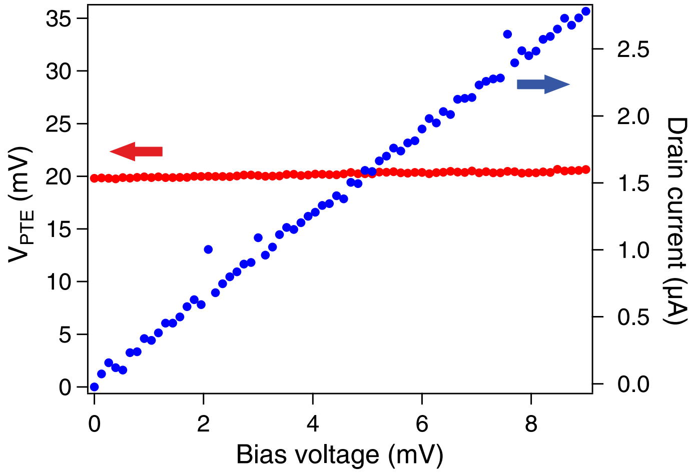

Supplementary Figure 6: Photovoltage (red) and source-drain current (blue) as a function of the graphene channel bias. We observe that the photocurrent remains constant, whereas the source-drain current increases linearly when increasing the bias voltage.

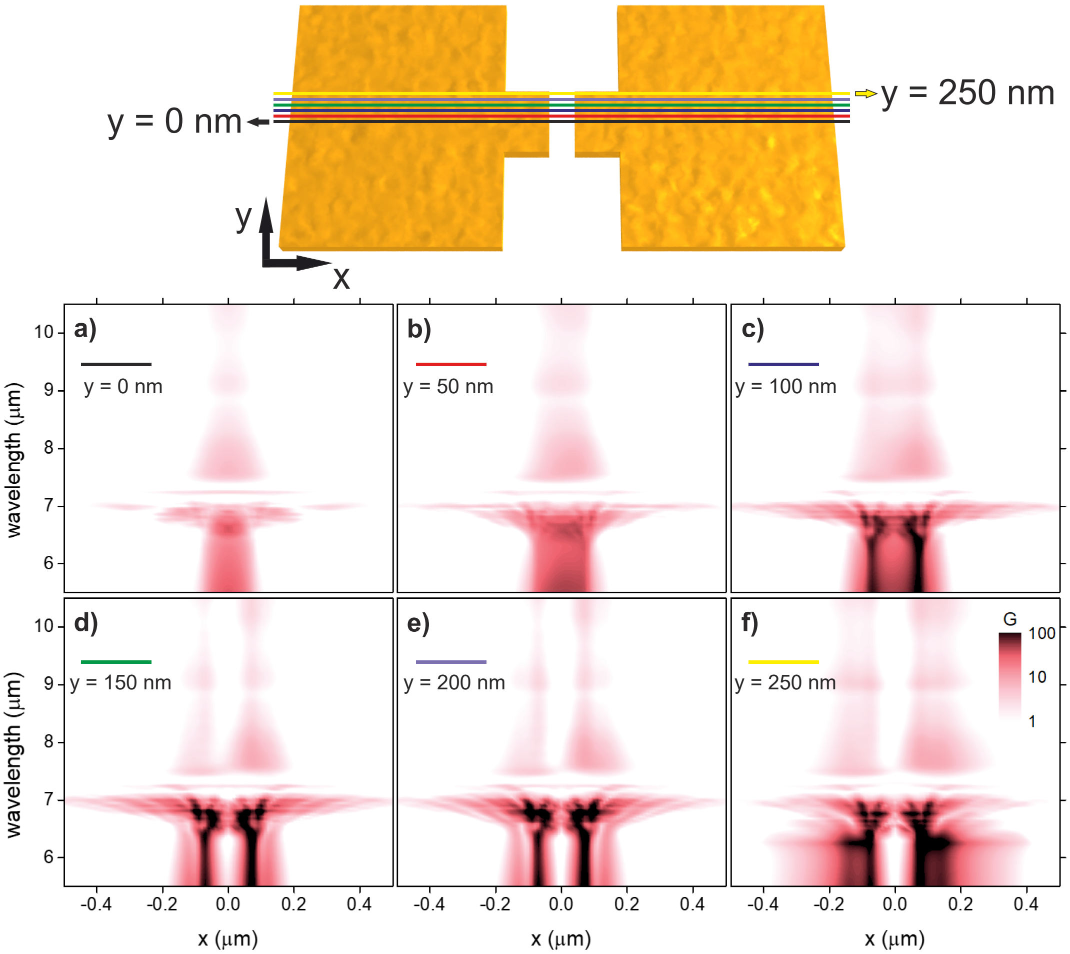

Supplementary Figure 7: G values for TM-polarization for different cross-sections across the gates along the source-drain direction (x direction, where x =0 is located at the center of the gate gap. This gap is 155 nm. See Fig. 1a-b in the main text for axis definition) as a function of wavelength. **a)** corresponds to a linecut of G across the center of the gates ($y = 0$) as indicated in the top schematic. Then, the rest indicate linecuts of G **b)** 50 nm, **c)** 100 nm, **d)** 150 nm, **e)** 200 nm and **f)** 250 nm above the the center of the gates. The gates and antenna overlap at $y > 100$ nm (or $y <$ -100 nm) away from the center ($y = 0$ nm). The upper antenna branch tip is located at $y =$ 100 nm. We observe that the spatial pattern of G varies with y and that G increases significantly where these two metal regions overlap ($y > 100$ nm).

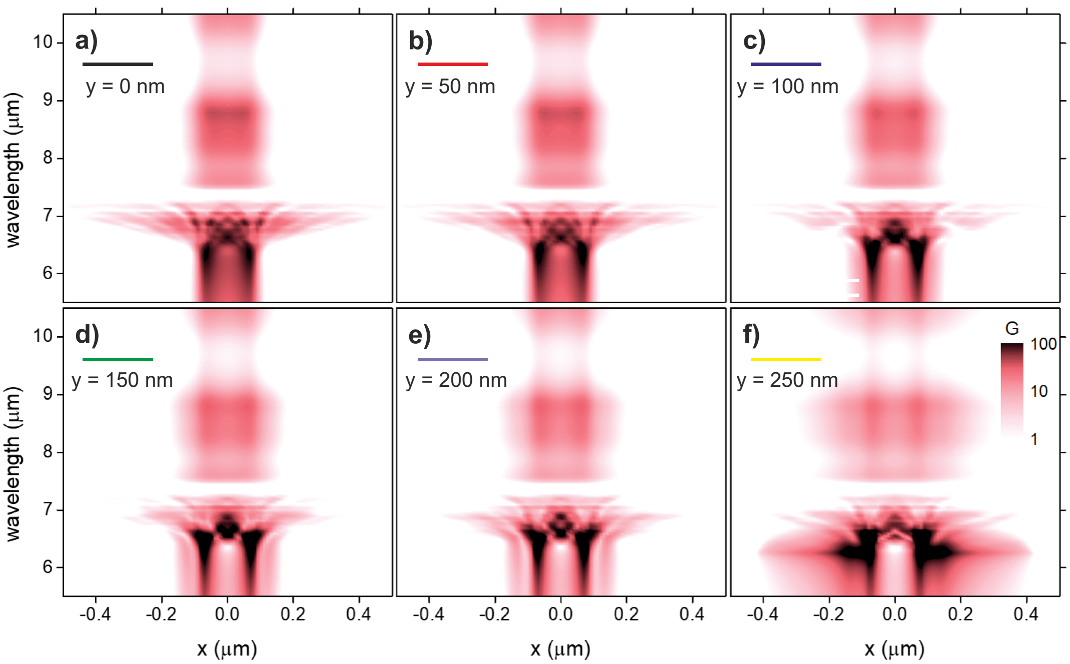

Supplementary Figure 8: Same as Supplementary Figure 7 but for TE-polarization. **a)** corresponds to a linecut of G across the center of the gates ($y = 0$). The rest indicate linecuts of G **b)** 50 nm, **c)** 100 nm, **d)** 150 nm, **e)** 200 nm and **f)** 250 nm above the the center of the gates. We observe that the spatial pattern of G varies while looking at it along different locations and that G greatly increases where the gate and antenna regions overlap ($y > 100$ nm).

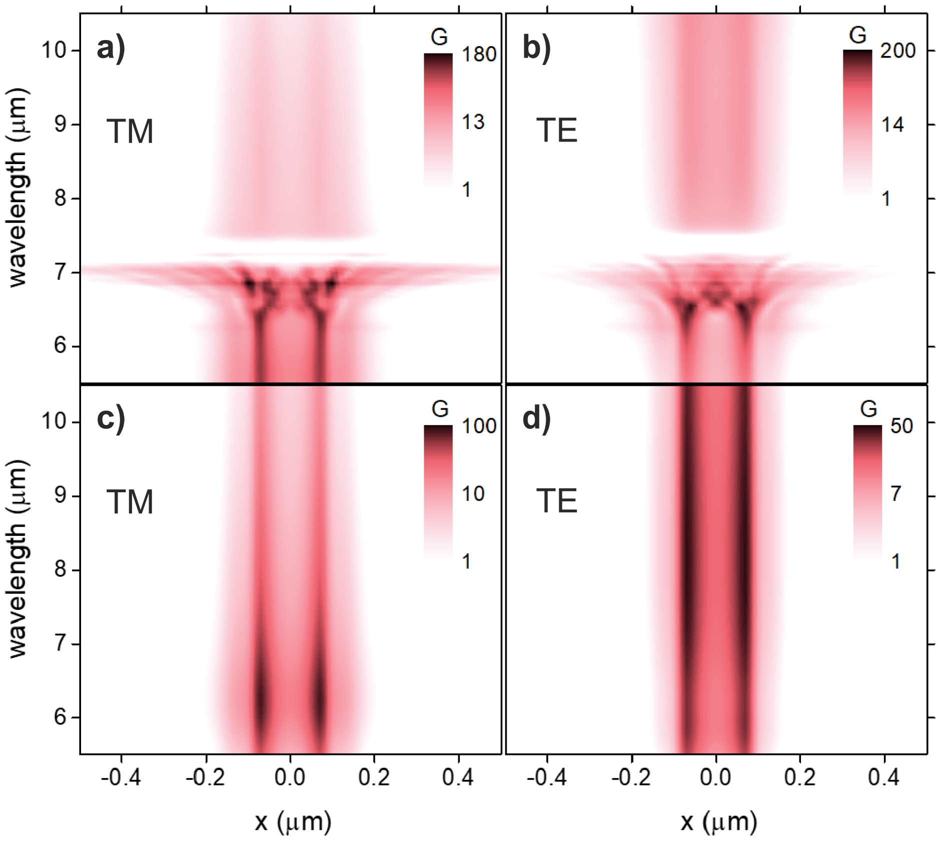

Supplementary Figure 9: Simulations of the absorption enhancement G along the source-drain direction (x direction and averaging over 500 nm in y direction, where $x = 0$ is located at the center of the gate gap. This gap is 155 nm. See Fig. 1a-b for axis definition) as a function of wavelength using different hBN and SiO_2 refractive indices configurations for TM (first column, **a, c**) and TE-polarization (second column, **b, d**). In **a)** and **b)**, we use the full dispersive optical model for the hBN but not for the SiO_2, for which we use a wavelength independent refractive index of $n = 1.5$. In **c)** and **d)**, we use wavelength-independent refractive indices for hBN ($n = 2.4$), SiO_2 ($n = 1.5$) and alumina ($n = 1.6$).

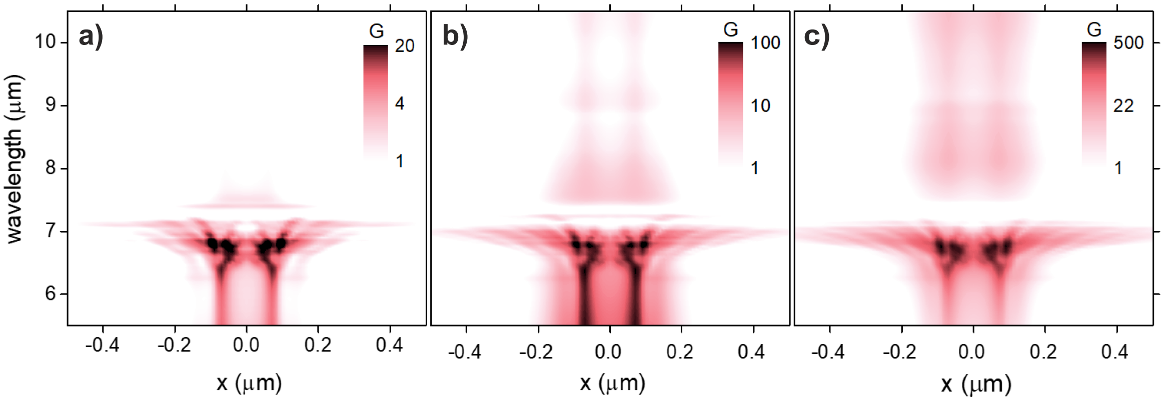

Supplementary Figure 10: 2D map of the simulated absorption enhancement G for TM-polarization along the source-drain direction (x direction and averaging over 500 nm in y direction, where $x = 0$ is located at the center of the gate gap. This gap is 155 nm. See Fig. 1a-b for axis definition) as a function of the wavelength (y-axis of the map) for total antenna lengths of **a)** $L = 1.8$ μm (non-resonant antenna within hBN RB), **b)** $L = 2.7$ μm (experimental/semi-resonant antenna within hBN RB) and **c)** $L = 4.8$ μm (resonant antenna within hBN RB). For longer antennas, we notice that the LSPR of the antenna is strongly coupled with the hBN HPPs as observed in the G map in **c**.

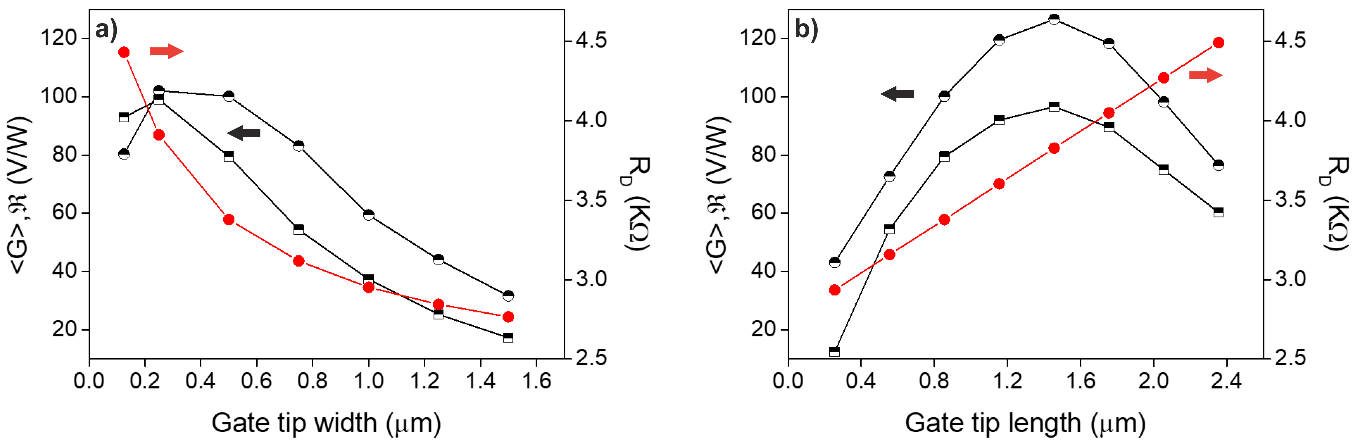

Supplementary Figure 11: Simulated responsivity ($\Re$ in V/W, shown in black circles), $<G>$ (defined in Supplementary Figure 3, black squares) and device resistance (R_D, shown in red circles) as a function of the geometric parameters of the H-shaped gates for TE-polarization at $\lambda = 6.5$ µm. **a)** Here we vary the tip width (W_{GT}). The gate tip length (L_{GT}) is fixed at 855 nm. We observe that W_{GT} correlates inversely with $<G>$. We attribute this trend to the fact that the plasmonic response of the local gates decreases with the increase of W_{GT} as shown in Supplementary Figure 3c. The only case that does not follow this trend is the case of W_{GT} =0.125 µm. In this case $<G>$ decreases because W_{GT} is smaller than the 0.2 µm gap between the branches of the bow-tie antenna, thus preventing the gates and antenna from overlapping, while the overlap region precisely contains the highest values of G as shown in Supplementary Figure 8. As shown in Supplementary Figure 5, lower $<G>$ values lead to smaller temperature gradients and consequently lower responsivities, since the responsivity is positively correlated with $<G>$. Finally, as W_{GT} increases the width of the doped graphene channel also increases leading to smaller R_D. The interplay between responsivity and R_D gives the optimum W_{GT} case as shown in Fig. 4b in the main text. **b)** Here we vary L_{GT}. W_{GT} is fixed at 500 nm. We observe that $<G>$ has a clear peak at L_{GT} = 1.455 µm. This trend is in excellent agreement with results in Supplementary Figure 3d-e, where the configuration using the same value of L_{GT} has the strongest response in the spectral position of the hBN upper RB. As in **a**, the responsivity positively correlates with $<G>$ and although R_D increases with L_{GT}, the optimum cases among all figures of merit (FOM) (Responsivity in V/W, A/W and NEP) remain roughly the same.

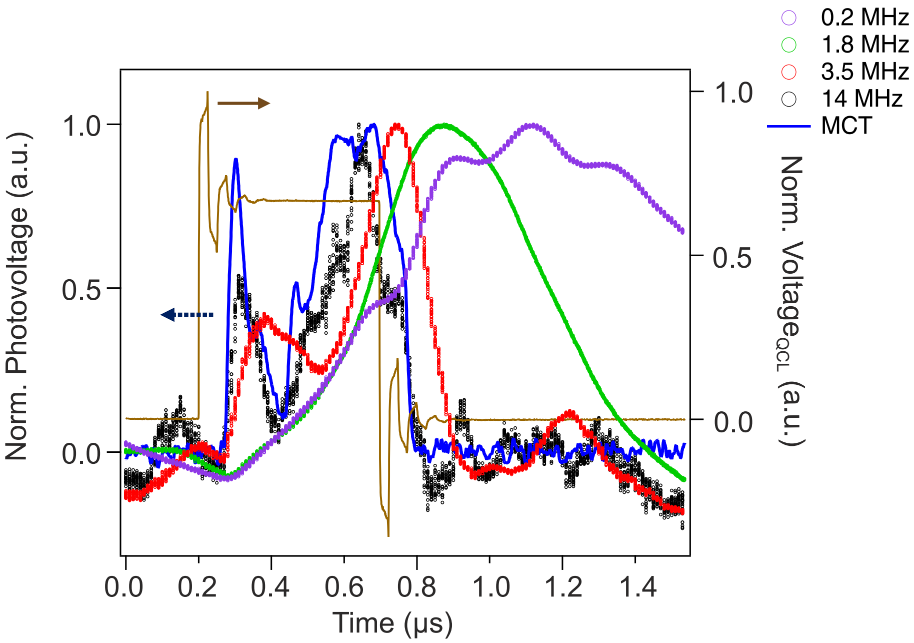

Supplementary Figure 12: Time-resolved photodetection traces for different operation bandwidths of the current amplifier. The speed increases (shorter rise time) with increasing bandwidth and the temporal response curve also resembles the photovoltage measured with the reference MCT detector as shown in blue solid line. The corresponding QCL voltage signal is shown in brown solid line.

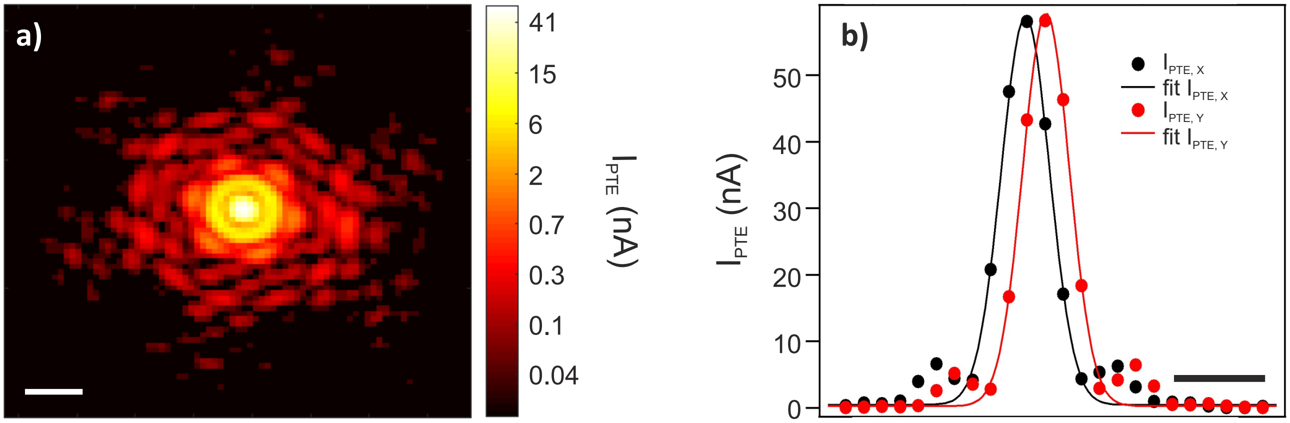

Supplementary Figure 13: Scanning photocurrent map at $\lambda = 6.6$ µm with TE-polarization. **a)** Scanning photocurrent map (log scale) over the x and y scan directions. We observe an Airy beam pattern consisting of a central spot followed by several rings that contain a very small fraction of the total input power ($P_{\text{in}} = 13.7$ µW, $P_{\text{diff}} = 2.1$ µW). The white scale bar stands for 20 µm. **b)** Linecuts of the map in **a**, showing I_{PTE} across the x (black) and y (red) direction. The scale bar corresponds to 10 µm. The dots represent the experimental I_{PTE} and the curves represent Gaussian fits. We obtain $w_{0,\text{x}} = 5.4$ µm and $w_{0,\text{y}} = 5.2$ µm. The maximum responsivity value achieved was 27 mA/W (92 V/W) which corresponds to a NEP of 82 pW/$\sqrt{\text{Hz}}$, with a noise spectral density of 2.21 pA/$\sqrt{\text{Hz}}$ for a device resistance of 3.38 kΩ at the *pn* junction configuration given by $E_{\text{F,L}} = 85$ meV, $E_{\text{F,R}} =$ -105 meV.

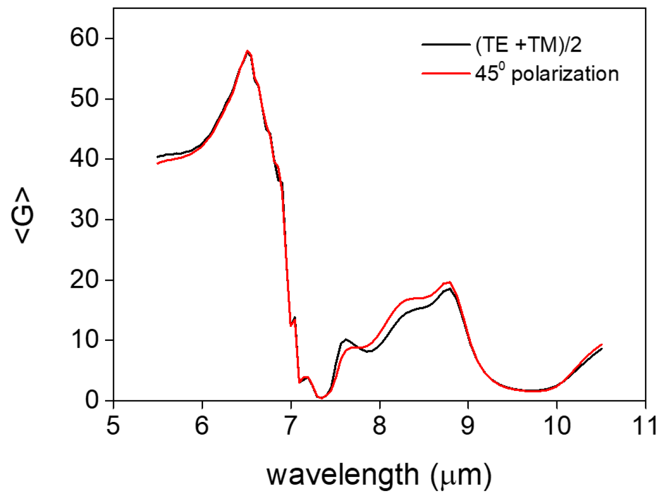

Supplementary Figure 14: Simulated absorption enhancement averaged over an area of 0.2×0.2 μm^2 around the device center ($x = y = 0$) for a 45 degree incident polarization respect to the bowtie antenna main axis (red curve) compared to the average TE and TM response (black curve). This result demonstrates that our system is linear and the photoresponse for an incident oblique polarization is the sum of the two components (TE and TM).

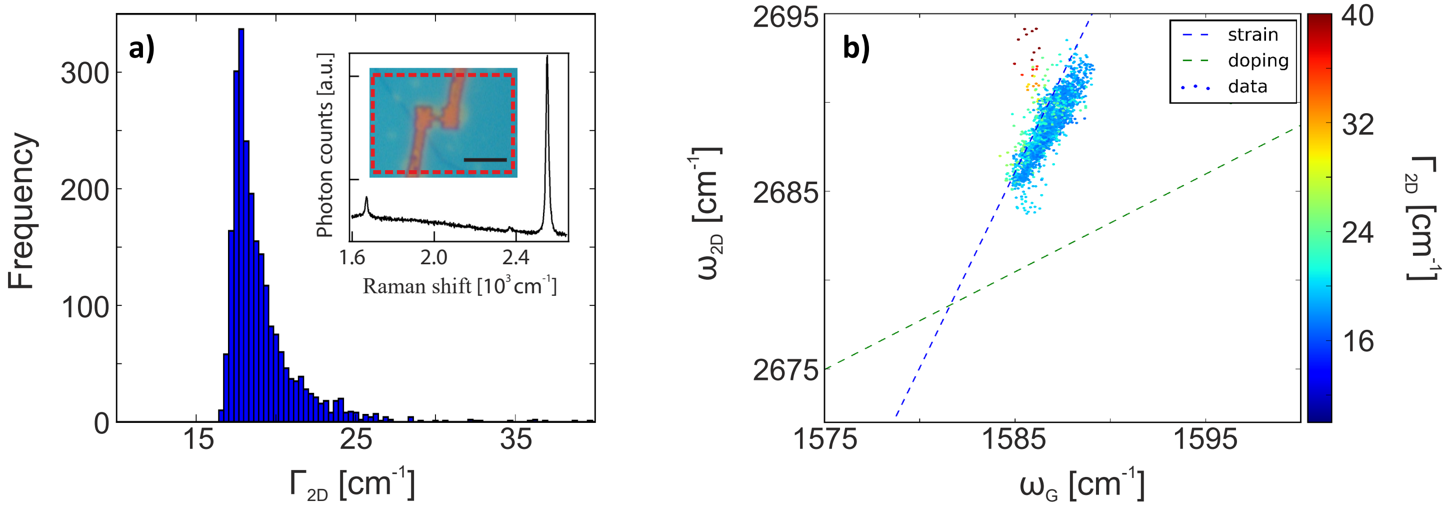

Supplementary Figure 15: Raman spectroscopy measurements of the 2D stack on top of the metal gates. **a)** Histogram plot of the full-width-half-maximum of the graphene 2D peak (Γ_{2D}) across a region of ∼13.5×9 μm^2 as shown in the inset in red dashed line. The inset scale bar corresponds to 4.2 μm. The mean Γ_{2D} is $\sim$ 18 cm^{-1}, which reflects the high quality of the monolayer graphene encapsulated in hBN. The inset plot corresponds to the usual spectrum obtained in these measurements for single layer graphene. **b)** 2D frequency peak (ω_{2D}) as a function of G frequency peak (ω_G) obtained from the Raman map in panel **a**, where the colorbar corresponds to the Γ_{2D} of the measured map. We obtained low doping values consistent with transport measurements (see Supplementary Figure 16) and modest strain values[17].

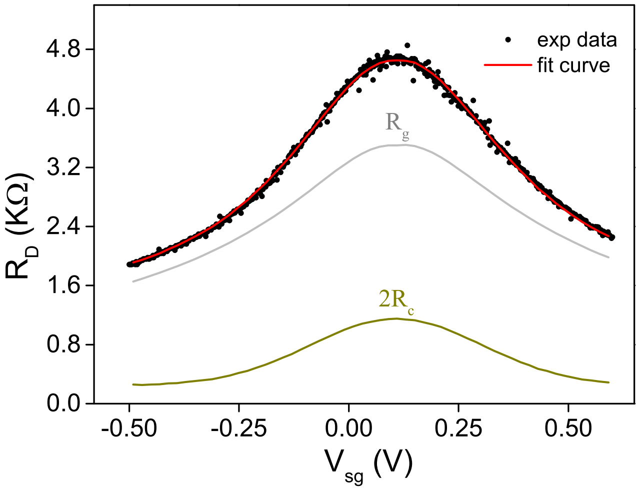

Supplementary Figure 16: Measured device resistance (R_{D}) as a function of the two gate voltages (V_{L} and V_{R}) both sweeping at the same voltage (V_{sg}). We fit the resistance curve using the model described in Supplementary Note 4. Contributions of both contact resistance ($2R_{\mathrm{c}}$) and graphene channel resistance (R_{g}) to R_{D} for all V_{sg} are also presented.

SUPPLEMENTARY DISCUSSION 1: GATE GAP EFFECT ON THE PHOTORESPONSE

We perform a set of simulations for the TM-polarization case changing the gate gap between 100 nm and 400 nm to observe its effect on the photoresponse performance. Specifically, as shown in Supplementary Figure 17a, the averaged spectral absorption enhancement profile $<G>$ is different for all cases, especially for the case of 400 nm gap where the peak values are much lower than the rest of the cases. This indicates that the interference patterns of hBN HPPs are affected by the gate gap. In terms of responsivity (Supplementary Figure 17b), these differences become more pronounced, as we can observe a clear optimum case for the 100 nm gap (the lower limit in terms of fabrication), while the performance of the 400 nm gap case is by far inferior. To elucidate this behaviour, we examine the spatial profiles of the parameters that affect the device performance, such as absorption (Supplementary Figure 18), hot electron temperature distribution (Supplementary Figure 20) and PTE voltage density (Supplementary Figure 22) for the cases of 100, 200, 300 and 400 nm gap at the wavelength where the maximum responsivity is observed for each case. Absorption profiles in Supplementary Figure 18 clearly explains the trends in Supplementary Figure 17a. As the overlap between the bowtie antenna and the tips of the gates decreases (by increasing the gap of the latter), both relative strength and spatial pattern is strongly altered. For 100 nm gap we observe large values of absorption, which however are concentrated beyond the gate edges where thermal conductivity is high (see Supplementary Figure 19). For 400 nm gap, on the other hand (no gate-antenna overlap) the absorption is strongly diminished but also more uniformly distributed inside the gap where thermal conductivity is low (see Supplementary Figure 19). These two conflicting attributes determine the temperature distribution as presented in Supplementary Figure 20. Indeed, as we increase the gap the differences we observe in peak temperature are smaller than those in absorption. Nonetheless, if we consider that for larger gaps the temperature peak is well centred within the gap area where the Seebeck coefficient (Supplementary Figure 21) has low values, we understand why PTE voltage generation diminishes for larger gaps, as is evident in Supplementary Figure 22. Taking also into account that resistance increases for larger gaps (see Supplementary Figure 23) explains the performance of our device as presented in Supplementary Figure 17b.

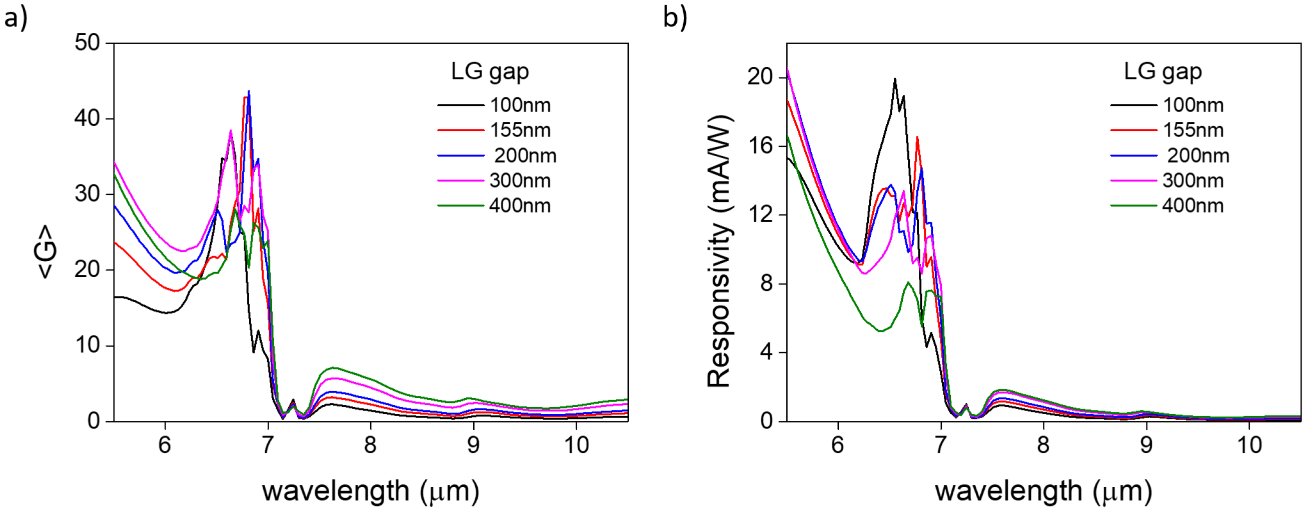

Supplementary Figure 17: a) Simulated $<G>$ (averaged over an area 0.5×0.5 μm^2 around the device center $x = y = 0$) for devices with different gate gaps with incident TM-polarization, **b)** corresponding spectral responsivity. The gate voltages configuration is $V_{\mathrm{L,R}} = \pm 0.5$ V ($E_{\mathrm{F}} = \pm 100$ meV).

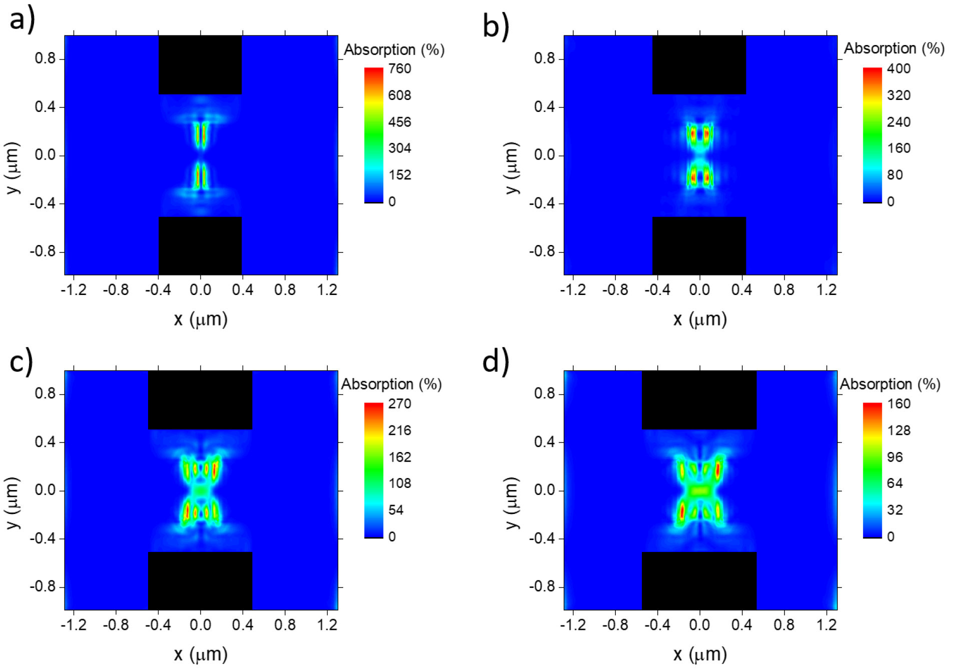

Supplementary Figure 18: Absorption distribution in graphene for TM-polarization with gate gap of **a)** 100 nm, **b)** 200 nm, **c)** 300 nm and **d)** 400 nm. The peak wavelength is chosen in each case (λ = 6.551, 6.767, 6.636 and 6.679 μm respectively). The gate voltage configuration is the same as displayed in Supplementary Figure 17.

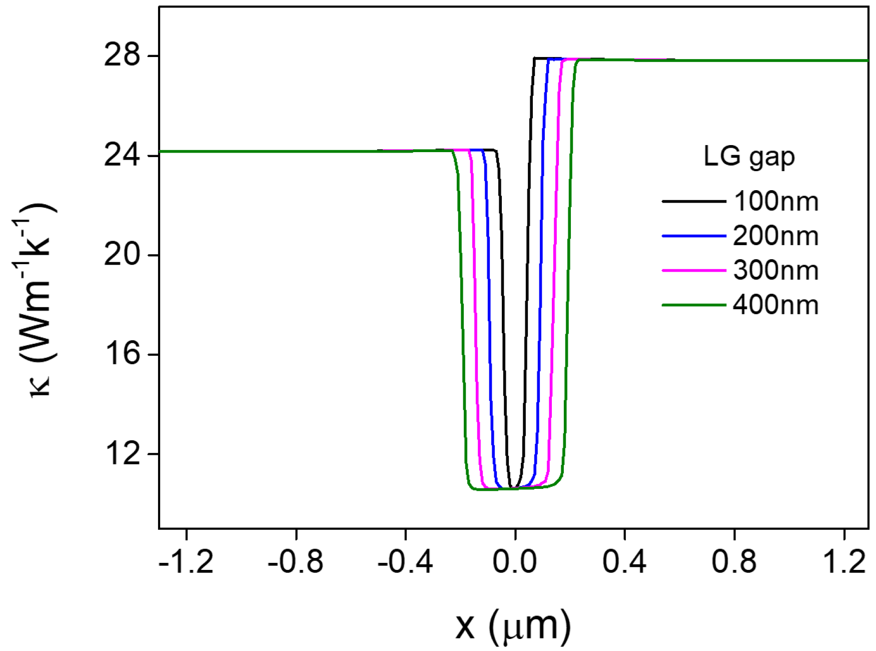

Supplementary Figure 19: Thermal conductivity across the graphene channel (at $y = 0$) for the devices with the different gate gaps and gate voltage configuration $V_{\mathrm{L,R}} = \pm 0.5$ V ($E_{\mathrm{F}} = \pm 100$ meV). The small imbalance is due to the difference in electron and hole mobilities.

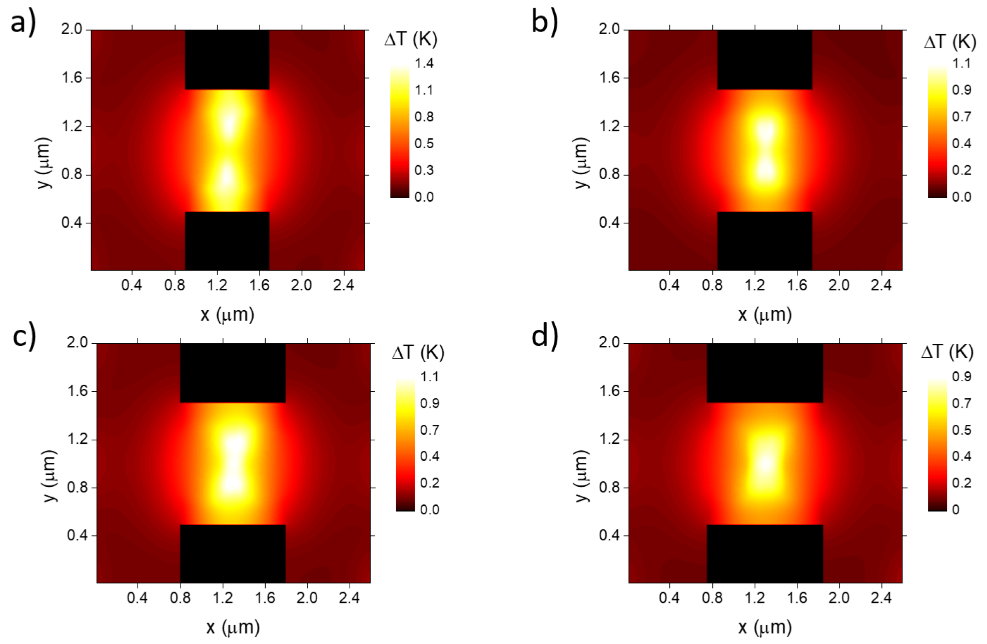

Supplementary Figure 20: Temperature distribution in graphene electrons for TM-polarization with gate gap of **a)** 100 nm, **b)** 200 nm, **c)** 300 nm and **d)** 400 nm. The peak wavelength chosen in each case is explained in Supplementary Figure 18. The gate voltages configuration is $V_{\mathrm{L,R}} = \pm 0.5$ V ($E_{\mathrm{F}} = \pm 100$ meV).

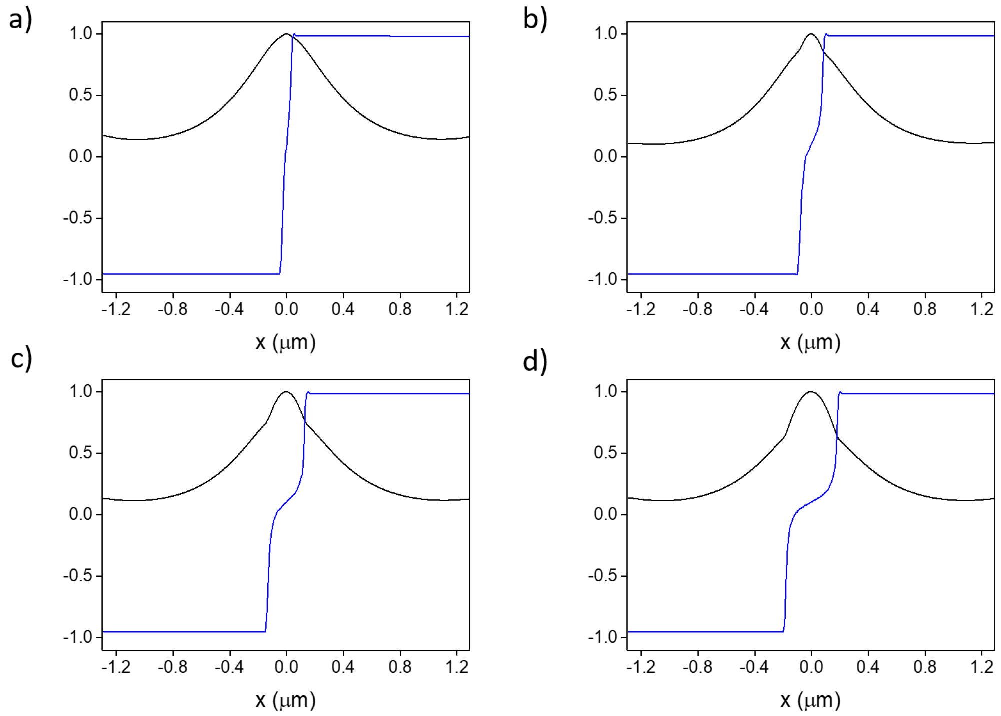

Supplementary Figure 21: Normalized ΔT (black lines) and Seebeck coefficient (blue lines) across the graphene channel (at $y = 0$) for TM-polarization with gate gap of **a)** 100 nm, **b)** 200 nm, **c)** 300 nm and **d)** 400 nm. The peak wavelength chosen in each case is explained in Supplementary Figure 18. The gate voltages configuration is $V_{\mathrm{L,R}} = \pm 0.5$ V ($E_{\mathrm{F}} = \pm 100$ meV).

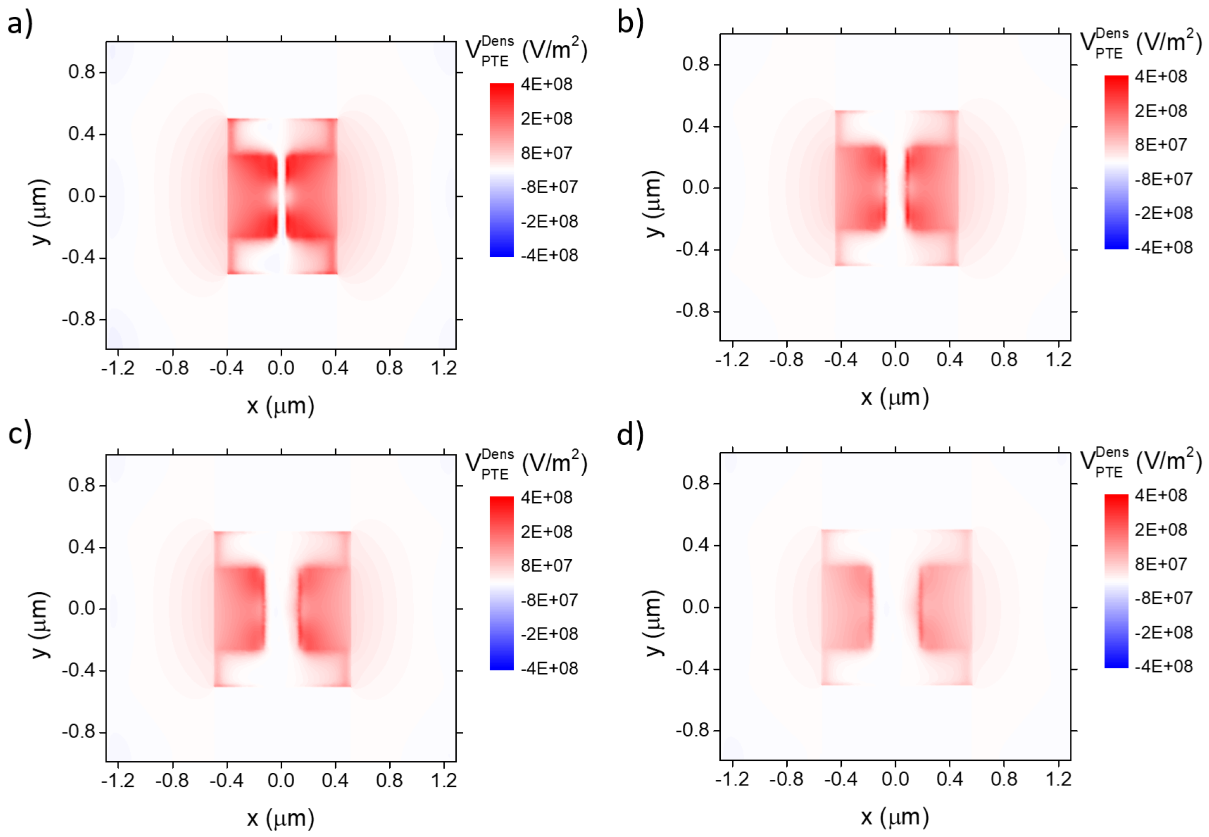

Supplementary Figure 22: PTE voltage density distribution in graphene for TM-polarization with gate gap of **a)** 100 nm, **b)** 200 nm, **c)** 300 nm and **d)** 400 nm. The peak wavelength chosen in each case is explained in Supplementary Figure 18. The gate voltages configuration is $V_{\mathrm{L,R}} = \pm 0.5$ V ($E_{\mathrm{F}} = \pm 100$ meV).

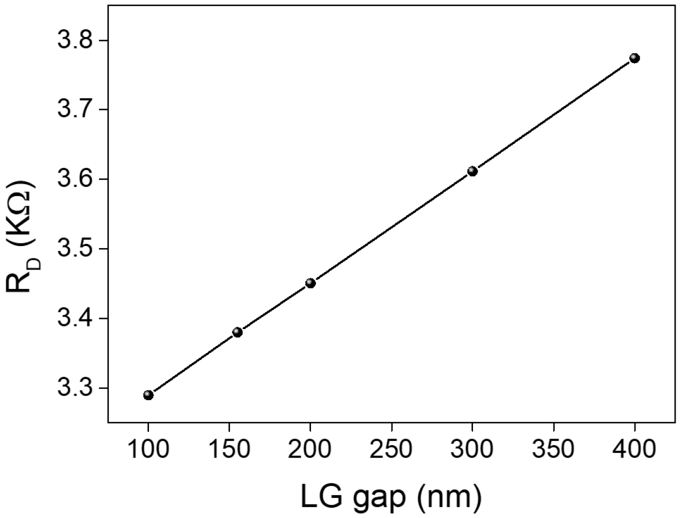

Supplementary Figure 23: Device resistance as function of the gate gap for the gate configuration $V_{\mathrm{L,R}} = \pm 0.5$ V ($E_{\mathrm{F}} = \pm 100$ meV).

SUPPLEMENTARY REFERENCES

[1] Woessner, A. et al. Highly confined low-loss plasmons in graphene–boron nitride heterostructures. *Nature Mater.* **14**, 421–425 (2015).

[2] Kischkat, J. et al. Mid-infrared optical properties of thin films of aluminum oxide, titanium dioxide, silicon dioxide, aluminum nitride, and silicon nitride. *Applied Optics* **51**, 6789–6798 (2012).

[3] Hanson, G. W. Quasi-transverse electromagnetic modes supported by a graphene parallel-plate waveguide. *Journal of Applied Physics* **104** (2008).

[4] Johnson, P. & Christy, R. Optical constants of the noble metals. *Phys. Rev. B* **6**, 4370–4379 (1972).

[5] Song, J. C. W., Rudner, M. S., Marcus, C. M. & Levitov, L. S. Hot carrier transport and photocurrent response in graphene. *Nano Letters* **11**, 4688–4692 (2011).

[6] Castilla, S. et al. Fast and Sensitive Terahertz Detection Using an Antenna-Integrated Graphene pn Junction. *Nano Letters* **19**, 2765–2773 (2019).

[7] Tielrooij, K.-J. et al. Out-of-plane heat transfer in van der Waals stacks through electron–hyperbolic phonon coupling. *Nature Nanotechnology* **13**, 41–46 (2018).

[8] Tan, Y. W. et al. Measurement of Scattering Rate and Minimum Conductivity in Graphene. *Phys. Rev. Lett.* **99**, 246803 (2007).

[9] Zuev, Y., Chang, W. & Kim, P. Thermoelectric and Magnetothermoelectric Transport Measurements of Graphene. *Phys. Rev. Lett.* **102**, 96807 (2009).

[10] Cutler, M. & Mott, N. F. Observation of anderson localization in an electron gas. *Physical Review* **181**, 1336–1340 (1969).

[11] Soavi, G. et al. Broadband, electrically tunable third-harmonic generation in graphene. *Nature Nanotechnology* **13**, 583–588 (2018).

[12] Kittel, C. Introduction to Solid State Physics (Wiley, 2004), 8 edn.

[13] McPherson, J. W., Kim, J., Shanware, A., Mogul, H. & Rodriguez, J. Trends in the ultimate breakdown strength of high dielectric-constant materials. *IEEE Transactions on Electron Devices* **50**, 1771–1778 (2003).

[14] Kim, K. K. et al. Synthesis and characterization of hexagonal boron nitride film as a dielectric layer for graphene devices. *ACS Nano* **6**, 8583–8590 (2012).

[15] Xia, F., Perebeinos, V., Lin, Y. M., Wu, Y. & Avouris, P. The origins and limits of metal-graphene junction resistance. *Nature Nanotechnology* **6**, 179–184 (2011).

[16] Mišković, Z. L. & Upadhyaya, N. Modeling electrolytically top-gated graphene. *Nanoscale Research Letters* **5**, 505–511 (2010).

[17] Lee, J. E., Ahn, G., Shim, J., Lee, Y. S. & Ryu, S. Optical separation of mechanical strain from charge doping in graphene. *Nature Communications* **3**, 1024–1028 (2012).
